# Substrate toxicity drives successive range expansions opposing spatial intermixing in cross-feeding consortia

**DOI:** 10.1093/ismeco/ycag085

**Published:** 2026-04-16

**Authors:** Xiaoli Chen, Miaoxiao Wang, Laipeng Luo, Liyun An, Xiaonan Liu, Yong Nie, Xiao-Lei Wu

**Affiliations:** School of Mechanics and Engineering Science, Peking University, Beijing 100871, China; Institute of Fundamental and Frontier Sciences, University of Electronic Science and Technology of China, Chengdu, Sichuan 611731, China; Microbial Systems Ecology Group, Institute of Biogeochemistry and Pollutant Dynamics, Department of Environmental Systems Sciences, ETH Zurich, 8006, Zurich, Switzerland; School of Mechanics and Engineering Science, Peking University, Beijing 100871, China; School of Biological Science and Technology, University of Jinan, Jinan, Shandong 250022, China; School of Mechanics and Engineering Science, Peking University, Beijing 100871, China; School of Mechanics and Engineering Science, Peking University, Beijing 100871, China; School of Mechanics and Engineering Science, Peking University, Beijing 100871, China; Institute of Ecology, Peking University, Beijing 100871, China

**Keywords:** spatial organization, cross-feeding, consortia, substrate toxicity, succession pattern, microbial diversity

## Abstract

Spatial organization plays a critical role in shaping microbial community structure and function, influencing ecological stability, resource utilization, and evolutionary dynamics. Microbial interactions such as competition and cooperation are key drivers of spatial patterning, yet the environmental factors modulating these interactions remain incompletely understood. Here, we investigated how toxic substrates influence the spatial organization of synthetic microbial communities engaged in metabolic cross-feeding. Using a synthetic *Pseudomonas stutzeri* consortium consisting of the detoxifier and consumer that cooperatively degrade the toxic compound salicylate, we found that increasing the substrate concentration leads to a distinct shift in spatial organization: the detoxifier increasingly dominates the outer periphery of the expanding colony, forming a “detoxifier-first” succession pattern. Mathematical modeling further revealed that this spatial arrangement emerges from substrate toxicity, which selectively favors the detoxifier. Substrate toxicity inhibits consumer proliferation. However, the detoxifier, capable of degrading the substrate, locally reduces toxicity and creates a protective microenvironment that enables nearby consumer cells to survive and grow. In return, the consumer provides essential final products that support the growth and expansion of the detoxifier. This reciprocal interaction establishes a directional dynamic in which the detoxifier, favored by its detoxification capability, colonizes first, paving the way for subsequent consumer proliferation. Our findings demonstrate that substrate toxicity is a crucial environmental factor shaping spatial organization and diversity in microbial communities. This study highlights the importance of considering both metabolic interactions and substrate properties in understanding microbial ecology.

## Introduction

Microbial communities typically inhabit spatially structured habitats and form high levels of spatial organization patterns [1–4]. Such spatial arrangements influence how microorganisms access resources, communicate, and interact, thereby shaping community assembly, dynamics, and ecosystem functions [5–8]. Microbial interactions, including competition and cooperation, can further modulate spatial organization, which in turn affects community stability, robustness, and functional performance [9–11]. The emergence of spatial structure is governed by both environmental constraints and biological processes [12], among which interspecific interactions are particularly critical [13, 14]. For example, competition for limited resources often results in spatial segregation [15, 16], whereas synergistic interactions tend to promote spatial intermixing, which facilitates species coexistence and maintains genetic diversity [17, 18].

Although synergistic consortia usually develop intermixed spatial patterns [17, 18], several natural examples deviate from this trend and exhibit spatial segregation among cooperative partners. For instance, in plaque, *Corynebacterium* and cocci form a spatially segregated corncob-like structure [19, 20], where *Corynebacterium* acts as a foundational taxon that structures the microenvironment to support cocci growth. Another notable exception to the commonly observed intermixing in synergistic microbial systems is found in anaerobic methanotrophic (ANME) consortia, where a syntrophic partnership between ANME archaea and sulfate-reducing bacteria gives rise to a shell-type spatial organization rather than intermixing for ANME-2 and ANME-3 aggregates [21, 22]. In these structures, archaea typically occupy the interior core, while the sulfate-reducing bacteria form an outer shell-like layer [23, 24]. Despite such extensive observations, the underlying mechanisms driving the emergence of these non-intuitive spatial organizations in synergistic microbial systems remain poorly understood.

One typical form of synergistic microbial interactions is the degradation of toxic substrates by metabolic cross-feeding [25], such as polycyclic aromatic hydrocarbons (PAHs) and their derivatives [26]. Previous studies have shown that consortia engaged in such synergistic degradation generally exhibit more intermixed spatial patterns than those formed under competitive conditions [27, 28]. However, toxic substrates often exhibit a dual role: they serve as carbon sources while simultaneously exerting toxic effects on microbial cells [29]. While synergistic utilization of these substrates is expected to promote intermixing, it remains unclear how varying substrate toxicity levels influence the spatial self-organization of synergistic microbial consortia. Moreover, the trade-off between nutrient benefit and toxicity stress may profoundly affect spatial dynamics, a topic that warrants further investigation.

**Figure 1:**
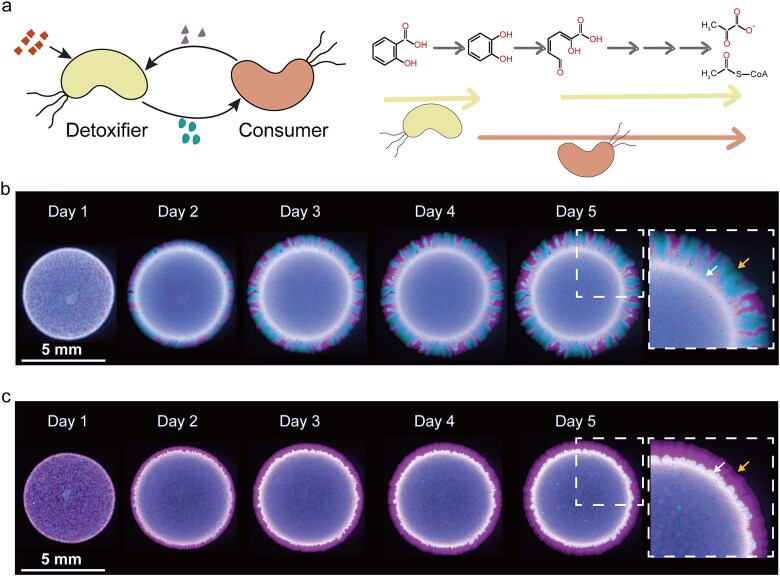
A two-strain synthetic cross-feeding microbial community was constructed and characterized. (a) In this system, the detoxifier strain converts the substrate (shown in red) into an intermediate (shown in green), which is then further processed by the consumer strain into final products (shown in purple) that support the growth of both strains. For (b) and (c), the detoxifier and consumer were mixed and inoculated onto agar plates supplemented with salicylate as the growth substrate for 5 days. The detoxifier expressing mCherry fluorescent protein is shown in magenta, while the consumer expressing eGFP fluorescent protein is shown in cyan. Dashed boxes mark regions that are enlarged to highlight the inoculum edge and the expansion frontier. Inoculum edges are indicated by white arrows, whereas expansion frontiers are indicated by yellow arrows. Different substrate concentrations produced distinct spatial patterns: (b) At a low substrate concentration (5 C-mM salicylate), we observed simultaneous expansion and segregation between the detoxifier and the consumer during colony expansion. (c) At a high substrate concentration (15 C-mM salicylate), a “detoxifier-first” pattern emerged, with the detoxifier leading the radial expansion and the consumer following behind the expansion front. Representative images from one of *n* = 3 biological replicates are shown.

To explore how substrate toxicity governs the spatial organization of cross-feeding microbial consortia, we employed a synthetic *Pseudomonas stutzeri* consortium engineered for salicylate degradation. In this system, two strains sequentially degrade salicylate, a compound known to be toxic to *P. stutzeri* [29]. The first strain (the detoxifier) converts salicylate into catechol. The second strain (the consumer) metabolizes catechol into final products (i.e. pyruvate and acetyl coenzyme A), which are secreted and support the growth of both strains (Fig. 1a). We combined mathematical modeling with spatial expansion experiments across a range of salicylate concentrations to investigate how toxic substrate governs spatial self-organization of metabolic cross-feeding communities. Our experimental results showed that high substrate concentration induced a successive range expansion, in which the detoxifier expanded first (primary expansion) and the consumer then followed (secondary expansion), opposing the spatial intermixing. Our model further provided mechanistic insights into the experimental observations and quantitatively described how substrate toxicity governs the spatial organization of cross-feeding consortia.

## Materials and methods

### Strains and plasmids

We used a previously developed synthetic microbial system for salicylate [30] and naphthalene [31] degradation. In the salicylate degradation biosystem, the detoxifier contains a deletion in *nahTH*, and can catalyze salicylate to catechol but cannot oxidize catechol to 2-hydroxymuconic-semialdehyde. The consumer contains a deletion in *nahG*, and can oxidize catechol to 2-hydroxymuconic-semialdehyde but cannot catalyze salicylate to catechol. In the naphthalene degradation biosystem, the detoxifier contains deletions in *nahG* and *nahTH*, and oxidizes naphthalene to salicylate but does not catalyze salicylate to 2-hydroxymuconic-semialdehyde. The consumer contains deletions in *nahA* and *nahC*, and catalyzes salicylate to 2-hydroxymuconic-semialdehyde but does not oxidize naphthalene to salicylate. The key functional genes involved in each metabolic step described above were expressed under an isopropyl β-D-1-thiogalactopyranoside (IPTG)-inducible P_lac_ promoter. Throughout this study, IPTG was maintained at a high and constant concentration (2 mM) to ensure stable expression of the detoxification and consumption pathways across all conditions. In spatially structured systems, metabolites are produced locally and diffuse gradually through the biofilm. Accordingly, secreted metabolites transiently accumulate in the immediate vicinity of producer cells, resulting in higher local concentrations prior to diffusion. To reduce the twitching motility [15] during colony expansion and lead to a much more defined spatial structure, all strains further contain a deletion in *pilAB*, which is required for type IV pilus [28]. To enable fluorescence microscopy, all strains were transformed by triparental filter mating with either plasmid pMMPc-egfp2 or plasmid pMMPc-mcherry2, constitutively expressing the enhanced green fluorescent protein (eGFP2) and the red fluorescent protein mCherry2, respectively [32]. Detailed methods are described in Supplementary Note 1. All strains, plasmids, and primers used in this study are described in Tables S1–S3.

### Growth conditions

Bacterial inocula were prepared from single colonies grown in rich medium and subsequently transferred to minimal medium before mixing detoxifier and consumer strains at defined ratios for colony expansion assays on agarose plates. Detailed incubation conditions are described in Supplementary Notes 2 and 3.

### Fluorescence imaging and image analysis

Colony expansion and spatial organization were visualized by fluorescence stereomicroscopy, and strain abundances and intermixing were quantified using custom image analysis pipelines. Detailed imaging settings and quantitative image analysis procedures are described in Supplementary Note 4.

### Flow cytometry

Population sizes and strain fractions were quantified by flow cytometry using fluorescent protein markers and bead-based normalization. Detailed flow cytometry protocols and data acquisition parameters are provided in Supplementary Note 5.

### Individual-based modeling

We constructed a two-dimensional model for the spatial expansion of cross-feeding consortia based on *gro* [33, 34]. The population dynamics and mass dynamics equations for both detoxifier and consumer were detailedly described in our previous study [30, 33] and in Supplementary Note 6. The detailed descriptions of all variables and parameters are available in Table S4 and Table S5. The assumptions in our IBM were as follows:

(i) In our simulation, only substrate as the sole carbon source is included in the metabolism since other sources are supplemented in nonlimiting concentrations. In the cross-feeding scenario, the substrate $S$ is converted into the intermediate $I$ locally solely by the detoxifier using enzyme ${E}_1$. The consumer produces the enzyme ${E}_2$ to break down $I$ into final product $P$ used for growth. The substrate conversion rate ${\nu}_S$ in the first reaction performed by the detoxifier (Equation (1)) and intermediate conversion rate ${\nu}_I$ in the second reaction performed by the consumer (Equation (2)) followed the Michaelis–Menten formulation:


(1) \begin{equation*} {\nu}_S=\frac{k_1{E}_1}{K_1+S}S \end{equation*}

(2) \begin{equation*} {\nu}_I=\frac{k_2{E}_2}{K_2+I}I \end{equation*}

${k}_i$ (*i* = 1 corresponding to the detoxifier; *i* = 2 corresponding to the consumer) is the specific rate of the *ith* reaction, ${K}_i$ (*i* = 1, 2) is Michaelis–Menten constant for the *ith* reaction.

(ii) The final product synthesized by the consumer can diffuse away for the detoxifier to obtain. The detoxifier depletes the final product as it grows. We employ the term “product” to mean any metabolites which can provide energy for cell growth, such as pyruvate and acetyl-CoA. The final product sustains the growth of both the detoxifier and the consumer through Monod’s equations. The growth rate for the *ith* cell ${g}_i$ is calculated as follows:


(3) \begin{equation*} {{g}}_{{i}}=\frac{{{kg}}_{{i}}{{P}}_{{i}}}{{{Kg}}_{{i}}+{{P}}_{{i}}} \end{equation*}

where ${kg}_i$ is the maximum growth rate, ${P}_i$ is product concentration, ${Kg}_i$ is Monod half-saturation coefficient. In addition, to mimic the experimental conditions, we add the specific pathway mechanisms to our basic IBM. For example, the growth rates of both populations are inhibited by local salicylate concentrations due to the toxicity of the salicylate [29, 35]. A Hill inhibitory function [36, 37] is used to model the effect of salicylate on bacterial growth rates $\Big(\frac{1}{1+{\left(\theta{S}_i\right)}^3}\Big)$. The growth dynamics of the detoxifier and the consumer follow the equation integrated with toxic terms [30]:


(4) \begin{equation*} {{g}}_{{i}}=\frac{{{kg}}_{{i}}{{P}}_{{i}}}{{{Kg}}_{{i}}+{{P}}_{{i}}}\bullet \frac{1}{1+{\left({\theta} {{S}}_i\right)}^3}-{d}_i\end{equation*}

## Results

### A toxic substrate with high concentrations drives successive range expansion toward a “detoxifier-first” pattern

To assess the effect of salicylate concentration on the spatial organization of cross-feeding microbial consortia (Fig. 1a), we inoculated the consortia composed of the detoxifier and the consumer at an initial ratio of 1:1 (OD_600_:OD_600_) and then deposited the mix onto the solid agarose medium containing salicylate as their sole carbon and energy source. To focus on the direct effects of substrate concentration on spatial expansion, both detoxifier and consumer contained a deletion in *pilAB*, which reduced the twitching motility during colony expansion and resulted in a much more spatially defined structure [15, 33] (Fig. S1). As shown in Fig. 1b–c, we observed two distinct spatial patterns depending on the substrate concentrations. At a low salicylate concentration (5 C-mM), the detoxifier and the consumer expanded simultaneously and became spatially segregated, transitioning from an initially well-mixed expansion front at inoculation to sector-like patterns over 5 days (Fig. 1b, Fig. S2). In contrast, at a high concentration (15 C-mM), the detoxifier occupied the expansion frontier, while the consumer lagged behind, a configuration referred to as the “detoxifier-first” pattern (Fig. 1c, Fig. S2). Time-lapse imaging from Day 1 to Day 5 further demonstrated a successive range expansion of the consortium, in which detoxifier cells advanced first, followed by the secondary expansion of consumer cells.

**Figure 2:**
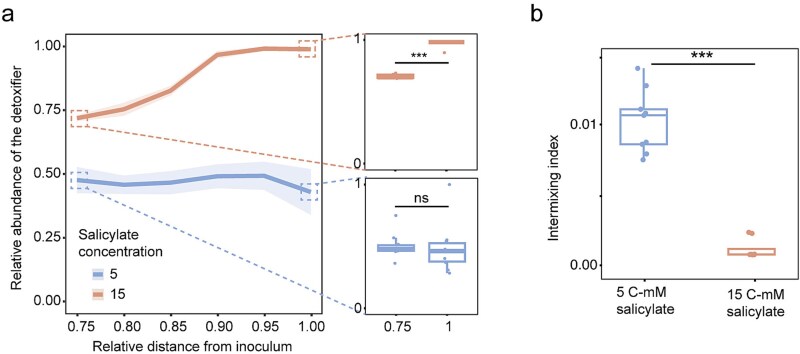
Effect of salicylate concentration on detoxifier abundance within colonies and community intermixing. (a) On the left panel, measurement of the relative abundance of the detoxifier from the edge of the inoculum to the expansion frontier, plotted as a function of radius. Lines and shaded areas correspond to the mean of data points and 95% confidence interval of the mean (*n* = 3), respectively. Specifically, under 5 C-mM salicylate, the relative abundance of the detoxifier at the edge of expansion frontier is similar to that at the edge of the inoculum (bottom right panel). In contrast, under 15 C-mM salicylate, the relative abundance of the detoxifier at the expansion frontier is significantly higher than at the edge of the inoculum (top right panel). (b) The effect of salicylate concentrations upon the intermixing index of communities at the edge of expansion frontier. The intermixing index under 5 C-mM salicylate was significantly higher than that under 15 C-mM salicylate (*n* = 3). *P* values were derived by *t*-test and shown as ns: P > .05; ^*^: .01 < *P* < .05; ^**^: .001 < *P* < .01; ^***^: *P* < .001.

We next quantified the relative abundance of the detoxifier from the edge of the inoculum to the frontier of the expansion zone. At a low salicylate concentration of 5 C-mM, the detoxifier fraction remained relatively constant across the expansion zone (Fig. 2a). In contrast, at a high salicylate concentration of 15 C-mM, the detoxifier fraction increased and then stabilized during radial expansion, resulting in a significantly higher proportion of detoxifier cells at the edge of the expansion compared to the edge of the inoculum (Fig. 2a, two-tailed two-sample *t*-test, *P* = 3.45 × 10^−14^). To evaluate how salicylate concentration influences local community diversity, we quantified the intermixing index at the colony periphery of the cross-feeding consortia. The intermixing index was significantly lower at 15 C-mM than at 5 C-mM (Fig. 2b, two-tailed two-sample *t*-test, *P* = 4.79 × 10^−7^), due to the successive expansion of the “detoxifier-first” pattern, which reduced spatial intermixing.

To examine whether the formation of the “detoxifier-first” pattern is robust to the initial strain ratio, we conducted colony range expansion experiments using not only an initial detoxifier-to-consumer ratio of 1:1, but also varied the initial ratios in a wider range (i.e. 1:2 and 2:1). We found that although altering the initial ratios affected the relative abundance of the detoxifier at a given radius, it did not alter the overall pattern: a low salicylate concentration (5 C-mM) drove simultaneous range expansion, whereas a high salicylate concentration (15 C-mM) led to sequential range expansion with the detoxifier expanding first (Fig. S3). Overall, our findings held true across initial detoxifier proportions ranging from 1:2 to 2:1. To investigate whether the distinct patterns observed at different salicylate concentrations were affected by the expression of different fluorescent proteins, we repeated the spatial self-organization experiments using strains with swapped fluorescent reporters. Consistent with our previous observations, a low salicylate concentration led to simultaneous expansion, whereas a high salicylate concentration resulted in sequential expansion, forming the characteristic “detoxifier-first” pattern (Fig. S4). The “detoxifier-first” pattern was robust, persisting across variations in initial inoculation ratios and fluorescent labeling schemes.

We then explored the possible drivers of the successive range expansion under the high substrate concentration. High substrate concentration increased the production of the final product, which serves as the direct carbon source for both the detoxifier and the consumer in the consortium [30]. In the absence of substrate toxicity, a strain with preferential access to or higher capacity for utilizing the final product would be expected to expand faster and dominate the expansion frontier. To test whether differences in final product utilization could account for the observed “detoxifier-first” pattern, we examined colony expansion when pyruvate, the final product of the cross-feeding pathway, was supplied directly as the sole carbon source. Under this condition, pyruvate induced segregation of the detoxifier and consumer into distinct sectors (Fig. S5a), a characteristic spatial pattern of competitive interactions commonly observed in bacterial range expansions [15, 17, 38]. Moreover, the consumer displayed a higher abundance than the detoxifier when grown on pyruvate (Fig. S5b, one-sample *t*-test, *P* = .0048, *n* = 3). Therefore, these results demonstrate that differential utilization of the final product cannot explain the emergence of the “detoxifier-first” pattern in the absence of substrate toxicity.

Based on these observations, we hypothesized that substrate toxicity drives the formation of the “detoxifier first” pattern. We examined the effect of salicylate toxicity on strain growth in spatially structured environments. We cultured the detoxifier and the consumer individually on agar plates supplemented with pyruvate and varying concentrations of salicylate. After 5 days of growth, we imaged the colonies (Fig. S6a) and further quantified colony cell numbers using flow cytometry. Our results showed that toxicity was minimal at low salicylate concentrations but increased at higher concentrations in both the detoxifier and the consumer, indicating that substrate toxicity is dose-dependent (Fig. S6b). Furthermore, at high salicylate concentrations, toxicity had asymmetric fitness effects: the detoxifier’s growth was less inhibited compared to the consumer’s. This is because the detoxifier can protect itself by converting salicylate into catechol, whereas the consumer cannot. As expected, the detoxifier better resisted the toxic substrate, as confirmed by our measurements. Increasing the initial substrate concentration potentially elevates substrate toxicity, which inhibits consumer proliferation. However, detoxifier cells capable of degrading salicylate mitigate this toxicity for nearby consumer cells. The consumer benefits from the detoxifier’s removal of the toxic substrate and, in return, supplies final products that support the growth and expansion of the detoxifier. This obligate mutualistic interaction gives rise to a succession pattern characterized by “detoxifier first.”

### Our individual-based model demonstrates that substrate toxicity is sufficient to produce the “detoxifier-first” pattern

Our experimental analysis above implicated that the “detoxifier-first” pattern was driven by substrate toxicity. To verify this mechanism, it was necessary to modulate the intrinsic toxicity of the substrate and gain insight into the dynamics of strain growth rates on the plates, which is experimentally challenging. Therefore, we developed an individual-based model to directly verify our hypothesis. To quantify colony expansion dynamics, which are crucial to understanding how substrate toxicity influences range expansion, we analyzed both the real-time ratio of the detoxifier’s growth rate to that of the consumer and the detoxifier’s abundance at the expanding periphery.

**Figure 3:**
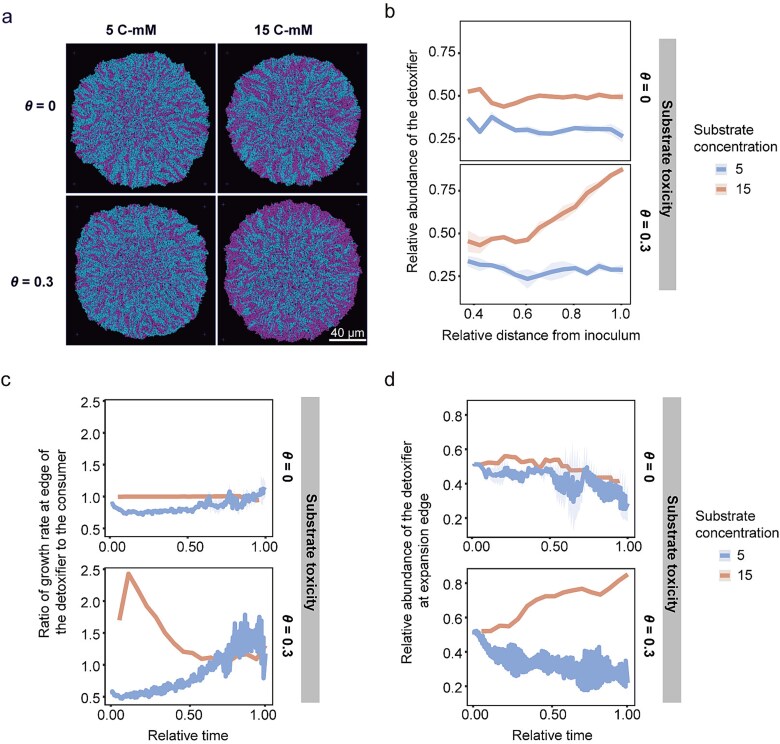
Computational modeling proved that substrate toxicity accounted for pattern diversification. (a) Simulating images with 5 C-mM and 15 C-mM salicylate as the sole carbon source in the absence ($\theta =0$) and presence of substrate toxicity ($\theta =0.3$) at the end of the simulation time. The final population size of the simulated colonies was ~8100 cells. Detoxifier cells are shown in magenta, while consumer cells are shown in cyan. (b) Measurement of relative abundance of the detoxifier from the edge of the inoculum toward the edge of expansion. At a high substrate concentration of 15 C-mM, the relative abundance of the detoxifier at the edge of the expansion frontier is similar to that at the edge of the inoculum in the absence of substrate toxicity ($\theta =0$). In contrast, the relative abundance of the detoxifier at the edge of the expansion frontier is significantly higher than at the edge of the inoculum in the presence of substrate toxicity ($\theta =0.3$). (c) Dynamics of the ratio of growth rate of the detoxifier to the consumer. (d) Dynamics of the relative abundance of the detoxifier at the edge of expansion. Lines and shaded areas correspond to the mean of data points and 95% confidence interval of the mean (*n* = 3), respectively.

To examine whether substrate toxicity shapes two distinct patterns at different substrate concentrations, we conducted simulations by varying the toxicity parameter in the inhibitory function $\frac{1}{1+{\left(\theta{S}_i\right)}^3}$ (see Methods for details). This functional form was derived from experimental measurements describing how substrate concentration influences strain growth (Fig. S6). In the absence of substrate toxicity ($\theta =0$), our modeling results showed that at a low substrate concentration of 5 C-mM, the consumer cells occupied more space and displaying a higher fraction at the colony edge (top left panel in Fig. 3a; the detoxifier abundance <0.5 along the blue line in the top panel of Fig. 3b). This observation occurred because the consumer had a higher growth rate than the detoxifier (the ratio of growth rate of the detoxifier to the consumer <1 along the blue line in the top panel of Fig. 3c). The higher growth rate was due to the low substrate concentration limiting the amount of product generated by the consumer. As a result, the consumer had preferential access to the product, supporting its faster growth compared to the detoxifier. When the substrate concentration increased to 15 C-mM, the consumer’s product consumption capacity became saturated, enabling the detoxifier to access a comparable amount of product. Therefore, both populations exhibited similar growth rates (top panel in Fig. 3c; the ratio of growth rate of the detoxifier to the consumer ≈ 1 along the orange line) and nearly equal fractions of each population at the colony edge during expansion (top right panel in Fig. 3a; the detoxifier abundance ≈ 0.5 along the orange line in the top panel of Fig. 3b; Y-axis value ≈ 0.5 along the orange line in the top panel of Fig. 3d).

In the presence of substrate toxicity ($\theta =0.3$, estimated from the inhibitory effect of salicylate on strain growth in Fig. S6), our model results were consistent with experimental observations (bottom panels in Fig. 3a; bottom panel in Fig. 3b). We found that at a low concentration of toxic substrate, the detoxifier exhibited a lower growth rate (the ratio of growth rate of the detoxifier to the consumer <1 along the blue line in the bottom panel of Fig. 3c) and a lower fraction at the colony edge (the Y-axis value <0.5 along the blue line in the bottom panel of Fig. 3d). In contrast, at a high concentration of toxic substrate, the relative abundance of the detoxifier at the expansion edge was consistently higher compared to a low concentration of 5 C-mM (bottom right panel in Fig. 3a; the bottom panel of Fig. 3d). As shown in the time-lapse simulation of the colony range expanision, the detoxifier primarily expanded and the consumer succeeded the primary expansion of the detoxifier (Fig. S7a–b), consistent with our experimental observations. In this scenario, localized substrate toxicity was much greater for the consumer than for the detoxifier (Fig. S7c), leading to a higher growth rate of the detoxifier relative to the consumer during colony range expansion (the ratio of growth rate of the detoxifier to the consumer >1 along the orange line in the bottom panel of Fig. 3c). Taken together, our simulation results demonstrated that excluding the effects of substrate toxicity, high substrate concentrations alone could not drive the formation of the “detoxifier first” pattern. In contrast, high substrate concentration and toxicity induced the successive range expansion, and shaped the “detoxifier first” pattern. These modeling results confirmed that toxicity arose from high substrate concentration, was responsible for the observed spatial organization of the “detoxifier first” pattern.

**Figure 4:**
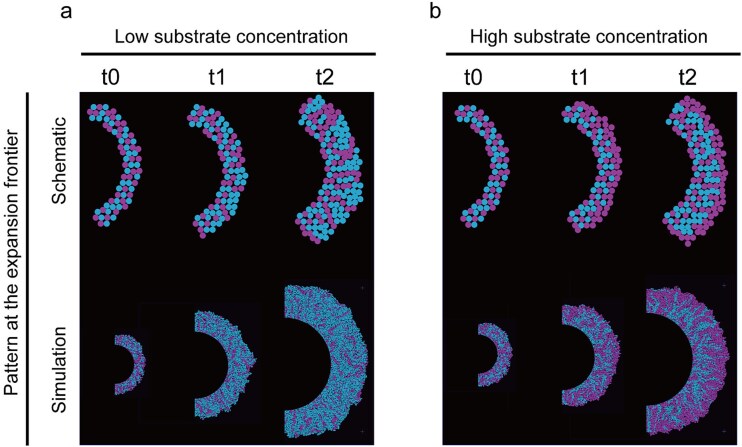
Schematic and simulation of spatial distribution of the cross-feeding community consisting of two strains during colony expansion. (a) Substrate does not have toxicity toward bacteria. (b) The substrate has toxicity to bacteria. In this case, the detoxifier cells expand first by the proliferating consumer cells to degrade the toxic substrate, resulting in sequential range expansion.

Our model elucidated the dynamics of the colony range expansion of a synergistic consortium in different concentrations of a toxic substrate: Prior to the onset of bacterial range expansion, the detoxifier and consumer cells are randomly distributed on the surface. At low substrate concentrations with negligible toxicity, the consumer exhibited a higher growth rate due to its preferential access to the final products synthesized by itself, leading to a higher abundance of the consumer than the detoxifier at the expanding periphery (Fig. 4a, Fig. S7a). In contrast, at high substrate concentrations, the consumer’s growth rate was lower than that of the detoxifier. This was because the detoxifier, rather than the consumer, was responsible for degrading the toxic substrate, creating significantly lower substrate concentrations surrounding the detoxifier compared to the consumer (Fig. 4b, Fig. S7b and Fig. S8). Consequently, the detoxifier with detoxification ability expanded first. The consumer near the detoxifier only began to grow after the detoxifier had sufficiently degraded the salicylate, reducing its local concentration. Once enough of the toxic substrate had been removed, the consumer cells started to proliferate and pushed the detoxifier cells outward as they expand, giving rise to the “detoxifier first” pattern (Fig. 4b).

To more thoroughly investigate the role of substrate toxicity, we performed additional simulations across a broader range of toxicity coefficients ($\theta$ = 0, 0.1, 0.3, 0.5) and substrate concentrations ($S$ = 5, 10, 15, 20 C-mM). We found that increasing substrate toxicity consistently enhanced the relative abundance of the detoxifier at the expansion frontier (Fig. S9), indicating a gradual transition toward the “detoxifier-first” pattern. Notably, at sufficiently high toxicity ($\theta$ = 0.5), the detoxifier dominated the expansion frontier with a relative abundance exceeding 0.9 even at a substrate concentration of $S$ = 10 C-mM, without requiring higher substrate concentrations (e.g. $S$ = 15 C-mM). These results demonstrate that increasing substrate toxicity shifts the emergence of the “detoxifier-first” pattern toward lower substrate concentrations.

To test whether substance diffusivity could account for the observed “detoxifier-first” pattern, we performed additional simulations across a broad range of diffusion coefficients ($D=$ 0.5, 1, 2, 5). In the absence of substrate toxicity, altering diffusivity and substrate concentration did not lead to detoxifier dominance at the expansion frontier. In contrast, when substrate toxicity was included ($\theta$ = 0.3) at a substrate concentration of 15 C-mM, the “detoxifier-first” pattern robustly persisted across all tested diffusion coefficients (Fig. S10), indicating that diffusion did not determine the emergence of the “detoxifier-first” pattern.

### The emergence of the “detoxifier-first” pattern is a general feature across other cross-feeding communities

**Figure 5:**
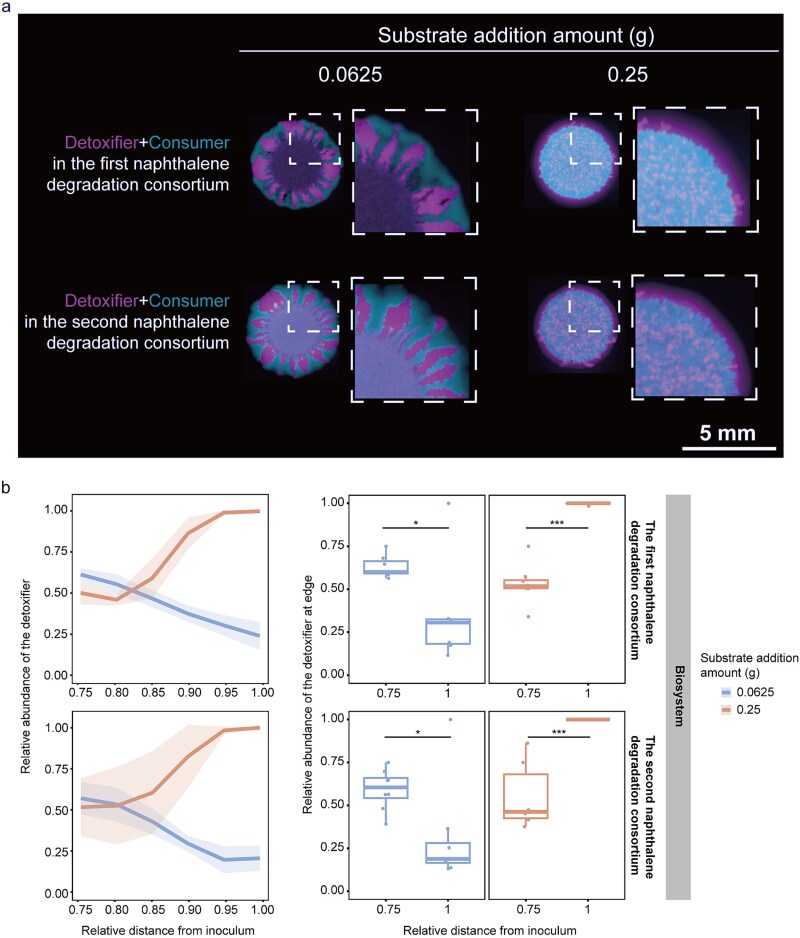
The effect of naphthalene addition amount on the pattern formation of cross-feeding consortia with detoxifier (magenta) and consumer (cyan). (a) Colonies were grown with naphthalene at a range of addition amounts for 21 days and imaged by fluorescence microscopy. (b) Measurement of relative abundance of the detoxifier from the edge of the inoculum toward the edge of expansion (left panels). Lines and shaded areas correspond to the mean of data points (*n* = 3) and 95% confidence interval of the mean, respectively. Under the condition with 0.25 g naphthalene as the carbon source, the relative abundance of the detoxifier at the edge of expansion frontier is significantly lower than that at the edge of the inoculum. However, under the condition with 1 g naphthalene as the carbon source, the relative abundance of the detoxifier at the edge of expansion frontier is significantly higher than that at the edge of the inoculum (right panels). Two-tailed two-sample *t-*test: *P* > .05 (ns); *P* < .05 (^*^); *P* < .01 (^**^); *P* < .001 (^***^).

Besides salicylate as the substrate, we employed two more cross-feeding microbial consortia for naphthalene degradation (Fig. S11). The experimental system consists of isogenic mutants from *P. stutzeri* AN10. In the first cross-feeding consortia, the detoxifier contains two loss-of-function deletions in both *nahG* and *nahTH* genes and can catalyze naphthalene but not salicylate. The consumer contains two loss-of-function deletions in both *nahA* and *nahC* genes and can catalyze salicylate but not naphthalene. In the second cross-feeding consortia, the detoxifier is the same as that in the first cross-feeding consortia. The consumer only contains a single loss-of-function deletion in the *nahA* gene and can catalyze both 1,2-hydroxy naphthalene and salicylate but not naphthalene. In both cross-feeding biosystems, the detoxifier and the consumer display cooperation when grown together with an exogenous supply of naphthalene as the substrate. At a low naphthalene level, we observed that the detoxifier and consumer expanded outward at approximately the same time, which fostered the formation of sectors during colony expansion (left column in Fig. 5a). In this scenario, the colony periphery was dominated by consumer cells (Fig. 5a–b). Increasing naphthalene levels increased the relative abundance of detoxifier at the frontier of the colony, giving rise to the emergence of the “detoxifier first” pattern (right column in Fig. 5a; Fig. 5b).

## Discussion

Combining synthetic bacterial consortia with computational simulations, our study provides mechanistic insight into the spatial self-organization of cross-feeding microbial communities under toxic environmental conditions. We propose a general principle governing the spatial expansion of microbial consortia on toxic substrates, which we term the “detoxifier-first” spatial succession. Specifically, at low substrate concentrations where toxicity is negligible, both the detoxifier and consumer expand simultaneously. In contrast, under high substrate concentrations, the detoxifier takes the spatial lead by first colonizing and detoxifying the surrounding environment, thereby reducing local substrate toxicity. This niche facilitation [39] enables the sensitive consumer to subsequently expand into the detoxified zone, and metabolize the intermediate compounds produced by the detoxifier. The emergent spatial pattern, characterized by detoxifier at the colony periphery and consumer in the inner region, represents a form of ecological succession driven by metabolic complementarity and asymmetrical toxicity resilience.

The spatial succession pattern where one population dominates the colony periphery has been observed in microbial synergistic denitrification [40, 41]. However, unlike the previously reported successive expansion driven by differences in lag times before nutrient consumption by community members [40], the emergent “detoxifier first” pattern in our system arises from the differing detoxification abilities of the detoxifier and consumer toward the toxic substrate. Unlike commonly used non-toxic substrates in previous spatial ecology studies, where increasing substrate concentration typically enhances growth and spatial mixing by mitigating the effects of genetic drift [15, 16], toxic substrates such as salicylate and naphthalene exert dual and opposing effects and trade-off. On the one hand, higher concentrations both enhance final product production that benefits the consumer; on the other hand, they simultaneously increase environmental toxicity that disproportionately inhibits the consumer relative to the detoxifier. In our system, the positive correlation between detoxifier abundance and substrate concentration indicates that toxicity is the dominant factor shaping spatial patterns.

Our work further shows that synergistic interactions are not a strict requirement for the emergence of the “detoxifier first” spatial pattern, but it enables stable coexistence of community members. In such systems, the mutualistic interaction ensures that both members persist: the detoxifier requires nutrients derived from the consumer, while the consumer depends on the detoxifier for detoxification. This bidirectional dependence naturally gives rise to a “detoxifier-first” successive range expansion along toxicity gradients, promoting long-term coexistence. Our previous studies have demonstrated that enhancing the detoxifier’s degradation capacity increased the relative abundance of the consumer [33]. In contrast, competitive systems comprising two non-cooperative strains (e.g. one capable and one incapable of detoxification) can also exhibit a similar peripheral dominance of the detoxifier under toxic conditions [42, 43]. In such cases, however, the peripheral dominance of the detoxifier arises solely from its protective effect, without reciprocal benefit. The survival of the detoxification-incapable strain depends entirely on its proximity to the detoxifier’s detoxification zone. If its fitness is substantially lower than that of the detoxifier, it is prone to be competitively excluded [44]. This pattern resembles that observed in antibiotic-resistant and sensitive microbial communities, where sensitive cells can temporarily coexist with resistant strains in environments containing antibiotics (β-lactams) through range-limited protection. However, in the absence of mutual dependence, the sensitive cells are usually eliminated if the resistant strain exhibits significantly higher fitness, ultimately preventing stable coexistence [45, 46]. Similar detoxification-driven spatial organization may also underlie the formation of shell-type structures observed in metabolically cooperative microbial consortia in natural environments, such as anaerobic oxidation of methane aggregates and shell-type structures observed in methanogenic granules. In these systems, shell-type architectures are thought to function as protective strategies against environmental toxicity, with outer-layer microorganisms removing inhibitory compounds. For example, sulfate-reducing bacteria located in the outer shell may shield more sensitive archaeal cells from harmful conditions by consuming inhibitory metabolites such as H_2_ and acetate, thereby mitigating toxicity to archaea and maintaining a favorable microenvironment that supports metabolic activity in the interior. This mechanism parallels our findings and suggests that detoxification-mediated chemical gradients may represent a general principle governing the emergence of non-intermixed spatial structures in cooperative microbial communities.

The term of cell’s growth rate (${g}_i=\frac{kg_i{P}_i}{Kg_i+{P}_i}\bullet \frac{1}{1+{\left(\theta{s}_i\right)}^3}-{d}_i$) is determined by several parameters involving the maximum consumption rate of the final product for cell growth ${kg}_i$, the toxicity of the substrate $\frac{1}{1+{\left(\theta{S}_i\right)}^3}$, and apparent maintenance rate ${d}_i$. When a 10-fold difference in ${kg}_i$ was introduced between two strains (with the detoxifier having a higher value than the consumer) in the absence of substrate toxicity, regardless of at the colony periphery or internally, the detoxifier dominates the colony and the “detoxifier first” pattern did not emerge. Similarly, when ${d}_i$ of the consumer was much higher than that of the detoxifier, the relative abundance of the detoxifier remained nearly constant across the colony radius, and the “detoxifier first” pattern disappeared (Fig. S12). Our *in silico* analyses indicated that in this model system, variation in ${kg}_i$ or ${d}_i$, without the presence of toxicity, did not lead to a shift from simultaneous to sequential expansion. This is because the influence of these parameters, ${kg}_i$ and ${d}_i$, on cell growth rates remained constant throughout the simulation, resulting in a fixed growth difference between the detoxifier and consumer, independent of substrate concentration. In contrast, the effect of substrate toxicity ($\frac{1}{1+{\left(\theta{S}_i\right)}^3}$) on growth rate varies with the concentration of substrate being degraded. Therefore, the emergent pattern of “detoxifier first” arises as a feedback response to the self-engineered landscape of substrate toxicity, which shapes the localized growth rates of both strains.

It is important to acknowledge several limitations of our study. First, due to the challenges of manipulating the toxicity of the substrate (salicylate), we were unable to experimentally verify the impact of substrate concentration on the spatial self-organization of microbial communities in the absence of substrate toxicity. Second, based on our modeling, two conditions are required for the emergence of the “detoxifier-first” spatial pattern: (i) the mass has an inhibitory effect on strain growth, and (ii) community members differ in their ability to degrade the toxic mass, resulting in distinct localized growth rates. Our findings suggest that the “detoxifier-first” pattern is not limited to cross-feeding systems; future experimental validation in other biosystems will be valuable.

## Supplementary Material

Supplementary_Information_ycag085

## Data Availability

The source codes used for our mathematical modelling are available at Github: https://github.com/chenxiaolili/gro-detoxifier_first.
