# Supplementary material for: Substrate toxicity drives successive range expansions opposing spatial intermixing in cross-feeding consortia
Source: ISME Commun. 2026 Apr 16;6(1):ycag085. doi: 10.1093/ismeco/ycag085 (PMC13137332; doi:10.1093/ismeco/ycag085)
Supplement: Supplementary_Information_ycag085 [file supplementary_information_ycag085.docx]

**Supplementary Materials for this manuscript include the following:**

Supplementary Notes 1-6

Supplementary Tables 1-5

Supplementary Figures 1-12

**Supplementary Notes**

# **Note 1 Triparental conjugation in *Pseudomonas stutzeri***

Triparental conjugation was used to introduce plasmids into *Pseudomonas stutzeri*. The donor strain (*Escherichia coli* Trans5α carrying the plasmid of interest), the recipient strain (*P. stutzeri*), and the helper strain (*E. coli* HB101 harboring the mobilization plasmid pRK2013) were first streaked on LB agar plates and incubated to obtain single colonies. Individual colonies were inoculated into 4 mL liquid medium in 25-mL culture tubes, with *E. coli* strains grown in LB supplemented with appropriate antibiotics and *P. stutzeri* grown in RB medium. *E. coli* cultures were incubated at 37°C with shaking at 220 rpm, and *P. stutzeri* cultures at 30°C and 200 rpm overnight. The overnight cultures were subsequently subcultured into fresh antibiotic-free medium (1% for *E. coli* and 2% for *P. stutzeri*) and grown to mid-exponential phase (OD_600_ ≈ 0.4). Cells from donor, recipient, and helper cultures were then harvested, washed with sterile saline solution (0.9% NaCl), and combined at a ratio of 1.8 mL donor, 1.2 mL recipient, and 0.8 mL helper. The mixed cell suspension was spotted onto antibiotic-free LB agar plates and incubated at 30°C overnight to allow conjugation. Cells were subsequently resuspended in sterile saline solution and plated on selective LB agar containing nalidixic acid (5 μg mL^-1^) and the appropriate antibiotic corresponding to the introduced plasmid. Plates were incubated at 30°C for about 36 h until *P. stutzeri* transconjugant colonies appeared. Correct transformants were verified by PCR using primers specific to the introduced plasmid.

**Note 2 Growth conditions**

To prepare the inoculum, frozen stocks of bacteria were streaked out on LB (5 g/L yeast extract, 10 g/L peptone, 5 g/L NaCl) agar Petri dishes, grown at 30^o^C for 48 h. Single clones of detoxifier and consumer were picked and grown separately overnight in RB liquid medium [1] (10 g/L yeast extract, 5 g/L beef extract, 10 g/L peptone, 5 g/L ammonium sulfate) under 30°C by shaking at 200 rpm and then inoculated into fresh RB at 2:100 dilution and grown to an OD_600_ (optical density at 600 nm) of 0.6. The cultures were washed three times with MF minimal medium to remove RB liquid medium by centrifuging at 3000 g for 5 min, resuspending in MF minimal medium [2] without carbon. The cell densities of the individual cultures were adjusted to an OD_600_ = 0.1. The cultures of the detoxifier and consumer were mixed according to the proportions required by experimental needs as described in the results section. The mixed cultures were vortexed briefly to ensure homogeneity. MF minimal medium was prepared by dissolving 17.9 g Na_2_HPO_4_·12H_2_O, 7.8 g NaH_2_PO_4_·2H_2_O, 5 g (NH_4_)_2_SO_4_, and 5 g KCl in ddH_2_O, supplemented with 10 mL SL-4 trace element solution, adjusted to pH 7.5 with NaOH, and brought to a final volume of 1 L. The medium was sterilized by autoclaving at 121°C for 20 min. The SL-6 trace element solution contained 0.1 g ZnSO_4_·7H_2_O, 0.03 g MnCl_2_·4H_2_O, 0.3 g H_3_BO_3_, 0.2 g CoCl_2_·6H_2_O, 0.01 g CuCl_2_·2H_2_O, 0.02 g NiCl_2_·6H_2_O, and 0.03 g Na_2_MoO_4_·2H_2_O dissolved in 1 L ddH_2_O. The SL-4 solution was prepared by dissolving 0.05 g EDTA and 0.02 g FeSO_4_·7H_2_O in ddH_2_O, followed by supplementation with 10 mL SL-6 solution to a final volume of 100 mL. Different concentrations of substrate were premixed with MF minimal medium. Agarose plates were prepared by pouring MF minimal medium with 1.5% agarose into 60-mm-diameter Petri dishes. A 1-μL drop of mixed cultures was pipetted on agarose plates that had dried for 8 h postpouring. Once the drops dried, the plates were overturned and incubated at 30°C for 5 days.

In experiments designed to decouple substrate toxicity from carbon availability, pyruvate was used as the sole carbon source because it represents the terminal product of the salicylate degradation pathway and does not introduce additional toxicity. Alternative intermediates in the pathway were not suitable for spatial expansion assays. Catechol does not support growth of the detoxifier and itself exerts toxic effects on cellular growth. Acetyl-coenzyme A is chemically unstable and rapidly hydrolyzes at the incubation temperature (30°C), rendering it incompatible with multi-day colony expansion experiments. Given that spatial expansion assays require incubation periods of up to five days, pyruvate provides the most stable and biologically relevant reference condition for isolating the effects of substrate toxicity from those of carbon availability.

# **Note 3 Colony pattern formation with vapor-phase naphthalene supplementation**

To prepare the culture plate, 5 mL MF minimal medium containing 1.5% agarose were poured in a Petri dish (6 cm in diameter) and left on the bench for 8 h before inoculation. Overnight cultures of two strains were adjusted to an OD_600_ of 0.1, mixed at a 1:1 ratio, vortexed briefly, and 1 μL of the mixed culture was spotted onto each agarose plate and allowed to dry for 15 min.

Because naphthalene has low solubility in water (~0.03 g L⁻¹ at room temperature), a 25% (w/v) naphthalene solution in acetone was prepared by dissolving 25 g naphthalene in 100 mL acetone and sterilized by filtration through a 0.22-μm filter. Aliquots of 0.25 mL (corresponding to 0.0625 g) or 1 mL (corresponding to 0.25 g) of this solution were evenly applied to 6-cm-diameter filter papers, which were then placed in the lids of 6-cm Petri dishes and left in a fume hood for approximately 30 min until complete evaporation of acetone, leaving solid naphthalene on the filter paper. Agarose plates inoculated with microbial consortia were inverted onto the corresponding Petri dish lids containing the naphthalene-loaded filter paper. The assembled plates were placed in sealed containers (MGC 7L, Japan) with a separate compartment containing sterile water to maintain humidity and prevent agarose desiccation. Plates were incubated at 30°C for 21 days, after which colony patterns were imaged using a Nikon SMZ800N stereomicroscope.

**Note 4 Fluorescence imaging and image analysis of colonies**

Colony patterns were imaged using a Nikon SMZ800N stereomicroscope equipped with an LED fluorescence imaging system. All images were taken at 10× magnification using a Nikon P-Plan Apo 1X/WF (W.D.70mm) objective. For imaging eGFP- and mCherry-tagged strains, AT515DC and AT635/60m filter cubes were used, respectively. Images were merged using FIJI software. Image analysis was performed with custom Wolfram Mathematica scripts. The procedure for quantifying the detoxifier’s relative abundance in colony patterns has been described elsewhere [3]. Briefly, each colored pixel was converted in a binary fashion to green or red. Then numbers of red and green pixels in a radial cross section of the colony were used to determine the relative abundance of each member [4]. Following previous studies [5, 6], the intermixing index was quantified on the binarized images with a circular windowing (Sholl-type) approach by counting red-green intersections along a circle of radius $r$. The intermixing index was then defined as $\frac{N_{r}}{{\pi r}/2}$ , where $N_{r}$ is the number of intersections at radius $r$. Data visualization was performed in R (www.r-project.org). The OD_600_ was used to measure population sizes and fluorescence intensities of eGFP and mCherry were measured to estimate the fraction of each strain in a community. Unless indicated otherwise, all simulations and experiments were performed with at least three biological replicates. For comparative statistics, the *P* values were obtained using an unpaired, two‐tailed, Student's t‐test.

**Note 5 Flow cytometry**

The entire bacterial colonies on agarose plates were scraped off and suspended in 1 mL saline solution (0.9% NaCl). Hereafter the samples were diluted to 10^-4^ - 10^-6^ cells/mL in saline solution. Each sample (200 µL) was supplemented with 20 µL of fluorescent bead stock (∼10^7^ /mL, GBW(E)120147, Haianhongmeng, China) as a reference to determine bacterial cell density. Bacterial cells were counted on a CytoFLEX S (Beckman, USA) flow cytometer. A 488 nm excitation laser and the FITC (525/40 nm filter) detector were used to detect eGFP. A 561 nm excitation laser and the ECD (610/20 nm filter) detector were used to detect mCherry. Samples were processed at a flow rate of 10 µL/min, until recording 2000 events for beads. CytExpert software was used to measure the fluorescent cells against the bead standard.

# **Note 6 Formulation, simulation, and analyses of our individual-based model**

We first built the formulations of our individual-based model describing substrate (Equation S1.1 and S1.2), intermediate (Equation S1.3 and S1.4), and product (Equation S1.5 and S1.6) concentrations in a two-step cross-feeding community [7]. The growth dynamics of the detoxifier and the consumer were modeled using a product-dependent Monod-type formulation (Equation S1.7-S1.10). Simulations were performed using the C++-based *gro* platform (https://github.com/liaupm/GRO-LIA) to model community expansion on a spatially structured two-dimensional plane of 160 μm × 160 μm. The simulation space was discretized into 1,600 grid units (each 4 μm × 4 μm) to represent limited diffusion of substances (S, I, and P). Mass diffusion was modeled according to Fick’s law with the default diffusivity coefficient set to 2, and the intracellular concentration of each substance was approximated by the concentration within the grid unit containing the corresponding cell. Simulations were initialized by randomly scattering and orienting a 1:1 consortium of detoxifier and consumer cells (1,500 cells in total) within a circular region of 30 μm in diameter. The simulation timestep was set to 0.01 min, and simulations were terminated when the total population size exceeded 8,100 cells. During each simulation, colony pattern images were generated at every time point using the built-in *gro* function Snapshot. Custom functions were incorporated into the *gro* code to record cell coordinates, mass concentrations, and population sizes for each cell type at each time point. Detailed parameter meanings were listed in Supplementary Table 4 and Supplementary Table 5.

$\frac{dS_{1}}{dt}=-\frac{k_{1}E_{1}}{K_{1}+S_{1}}S_{1}\cdot V_{1}+D_{S}\cdot{\nabla^{2}S}_{1}$ [S1.1]

$\frac{dS_{2}}{dt}=-D_{S}\cdot{\nabla^{2}S}_{2}$ [S1.2]

$\frac{dI_{1}}{dt}=\frac{k_{1}E_{1}}{K_{1}+S_{1}}S_{1}\cdot V_{1}-D_{I}\cdot{\nabla^{2}I}_{1}$ [S1.3]

$\frac{dI_{2}}{dt}=D_{I}\cdot{\nabla^{2}I}_{2}-\frac{k_{2}E_{2}}{K_{2}+I_{2}}I_{2}\cdot V_{2}$ [S1.4]

$\frac{dP_{1}}{dt}=D_{P}\cdot{\nabla^{2}P}_{1}-\frac{{kg}_{1}P_{1}}{{Kg}_{1}+P_{1}}P_{1}$ [S1.5]

$\frac{dP_{2}}{dt}=\frac{k_{2}E_{2}}{K_{2}+I_{2}}I_{2}\cdot V_{2}-\frac{{kg}_{2}P_{2}}{{Kg}_{2}+P_{2}}P_{2}-D_{P}\cdot{\nabla^{2}P}_{2}$ [S1.6]

$V_{1}=\frac{4}{3}\pi\cdot{(\frac{w}{2})}^{3}+\pi\cdot{(\frac{w}{2})}^{2}\cdot L_{1}$ [S1.7]

$V_{2}=\frac{4}{3}\pi\cdot{(\frac{w}{2})}^{3}+\pi\cdot{(\frac{w}{2})}^{2}\cdot L_{2}$ [S1.8]

$\frac{dL_{1}}{dt}=(\frac{{kg}_{1}}{{Kg}_{1}+P_{1}}P_{1}\cdot Y_{1}\cdot C_{1}$ $-d_{1})\cdot L_{1}$ [S1.9]

$\frac{dL_{2}}{dt}=(\frac{{kg}_{2}}{{Kg}_{2}+P_{2}}P_{2}\cdot Y_{2}\cdot C_{2}$ $-d_{2})\cdot L_{2}$ [S1.10]

Table 1 Sources of the strains used in this study

| Strain | Features | Source |
| --- | --- | --- |
| *E.coli* Trans5α | F^-^φ80 *lac ZΔM15Δ(lacZYA-arg F) U169 endA1 recA1* *hsdR17(*r_k_^-^, m_k_^+^*) supE44λ^-^ thi -1 gyrA96 relA1 phoA* | TransGen |
| *E.coli* HB101 | F^-^ *mcrB mrr hsdS20*(rB mB) *recA13 leuB6 ara-14 proA2* *lacY1 galK2 xyl-5 mtl-1 rpsL20*(Smr) *glnV44* | Chao Gao^1^ |
| *P. stutzeri* AN0000 | AN1111Δ*nahA::G*Δ*nahC::G*Δ*nahG::T*Δ*nahTH::A* | Miaoxiao Wang [3] |
| *P. stutzeri* AN0001 | AN1111Δ*nahA::A*Δ*nahC::C*Δ*nahG::G* | Miaoxiao Wang [3] |
| *P. stutzeri* AN0010 | AN1111Δ*nahA::T*Δ*nahC::A*Δ*nahTH::C* | Miaoxiao Wang [3] |
| *P. stutzeri* AN1100 | AN1111Δ*nahG::T*Δ*nahTH::A* | Miaoxiao Wang [8] |
| *P. stutzeri* AN0011 | AN1111Δ*nahA::G*Δ*nahC::G* | Miaoxiao Wang [8] |
| *P. stutzeri* AN0001-eGFP2 | AN0001 containing pMMPc-eGFP2 | This study |
| *P. stutzeri* AN0010-mCherry2 | AN0010 containing pMMPc-mCherry2 | This study |
| *P. stutzeri* AN0001ΔpilAB | Deletion of *pilA* and *pilB* in *P. stutzeri* AN0001 | Miaoxiao Wang [3] |
| *P. stutzeri* AN0010ΔpilAB | Deletion of *pilA* and *pilB* in *P. stutzeri* AN0010 | Miaoxiao Wang [3] |
| *P. stutzeri* AN1100ΔpilAB | Deletion of *pilA* and *pilB* in *P. stutzeri* AN1100 | This study |
| *P. stutzeri* AN0011ΔpilAB | Deletion of *pilA* and *pilB* in *P. stutzeri* AN0011 | This study |
| *P. stutzeri* AN0001ΔpilAB-eGFP2 | *P. stutzeri* AN0001ΔpilAB containing pMMPc-eGFP2 | This study |
| *P. stutzeri* AN0001ΔpilAB-mCherry2 | *P. stutzeri* AN0001ΔpilAB containing pMMPc-mcherry2 | This study |
| *P. stutzeri* AN0010ΔpilAB-eGFP2 | *P. stutzeri* AN0010ΔpilAB containing pMMPc-eGFP2 | This study |
| *P. stutzeri* AN0010ΔpilAB-mCherry2 | *P. stutzeri* AN0010ΔpilAB containing pMMPc-mcherry2 | This study |
| *P. stutzeri* AN0011ΔpilAB-eGFP2 | *P. stutzeri* AN0011ΔpilAB containing pMMPc-eGFP2 | This study |
| *P. stutzeri* AN0011ΔpilAB-mCherry2 | *P. stutzeri* AN0011ΔpilAB containing pMMPc-mcherry2 | This study |
| *P. stutzeri* AN1100ΔpilAB-eGFP2 | *P. stutzeri* AN1100ΔpilAB containing pMMPc-eGFP2 | This study |
| *P. stutzeri* AN1100ΔpilAB-mCherry2 | *P. stutzeri* AN1100ΔpilAB containing pMMPc-mcherry2 | This study |

Table 2 Sources of the plasmids used in this study

| Plasmid | Features | Source |
| --- | --- | --- |
| pRK2013 | Helper plasmid for conjugation; Km^R^ | Lab of Professor Lin Min, CAAS |
| pMMPc-Gm | Shuttle vector between *E. coli* and *P. stutzeri* | Lab of Professor Ping Xu, SJTU |
| pMMPc-eGFP2^*^ | *eGFP* gene on pMMPc-Gm | This study |
| pMMPc-mCherry2^*^ | *mcherry* gene on pMMPc-Gm | This study |

^*^Sequences of plasmids constructed in this study are provided below.

1. pMMPc-eGFP2 ttcaaagaaggagatatacatATGGTGTCGAAGGGCGAAGAACTGTTCACCGGCGTGGTGCCGATCCTGGTGGAACTGGACGGCGACGTGAACGGCCACAAGTTCTCGGTGTCGGGCGAAGGCGAAGGCGACGCCACCTACGGCAAGCTGACCCTGAAGTTCATCTGCACCACCGGCAAGCTGCCGGTGCCGTGGCCGACCCTGGTGACCACCCTGACCTACGGCGTGCAGTGCTTCTCGCGCTACCCGGACCACATGAAGCAGCACGACTTCTTCAAGTCGGCCATGCCGGAAGGCTACGTGCAGGAACGCACCATCTTCTTCAAGGACGACGGCAACTACAAGACCCGCGCCGAAGTGAAGTTCGAAGGCGACACCCTGGTGAACCGCATCGAACTGAAGGGCATCGACTTCAAGGAAGACGGCAACATCCTGGGCCACAAGCTGGAATACAACTACAACTCGCACAACGTGTACATCATGGCCGACAAGCAGAAGAACGGCATCAAGGTGAACTTCAAGATCCGCCACAACATCGAAGACGGCTCGGTGCAGCTGGCCGACCACTACCAGCAGAACACCCCGATCGGCGACGGCCCGGTGCTGCTGCCGGACAACCACTACCTGTCGACCCAGTCGGCCCTGTCGAAGGACCCGAACGAAAAGCGCGACCACATGGTGCTGCTGGAATTCGTGACCGCCGCCGGCATCACCCTGGGCATGGACGAACTGTACAAGTAAaagcttttaggtggcggtacttgggtcgatatcaaagtgcatcacttcttcccgtatgcccaactttgtatagagagccactgcgggatcgtcaccgtaatctgcttgcacgtagatcacataagcaccaagcgcgttggcctcatgcttgaggagattgatgagcgcggtggcaatgccctgcctccggtgctcgccggagactgcgagatcatagatatagatctcactacgcggctgctcaaacttgggcagaacgtaagccgcgagagcgccaacaaccgcttcttggtcgaaggcagcaagcgcgatgaatgtcttactacggagcaagttcccgaggtaatcggagtccggctgatgttgggagtaggtggctacgtctccgaactcacgaccgaaaagatcaagagcagcccgcatggatttgacttggtcagggccgagcctacatgtgcgaatgatgcccatacttgagccacctaactttgttttagggcgactgccctgctgcgtaacatcgttgctgctgcgtaacatcgttgctgctccataacatcaaacatcgacccacggcgtaacgcgcttgctgcttggatgcccgaggcatagactgtacaaaaaaacagtcataacaagccatgaaaaccgccactgcgccgttaccaccgctgcgttcggtcaaggttctggaccagttgcgtgagcgcatacgctacttgcattacagtttacgaaccgaacaggcttatgtcaattcgatccattgctgttgacaaagggaatcagcgatcggaggaccgaaggagctaaccgcttttttgcacaacatgggggatcatgtaactcgccttgatcgttgggaaccggagctgaatgaagccataccaaacgacgagcgtgacaccacgatgcctgtagcaatggcaacaacgttgcgcaaactattaactggcgaactacttactctagcttcccggcaacaattaatagactggatggaggcggataaagttgcaggaccacttctgcgctcggcccttccggctggctggtttattgctgataaatctggagccggtgagcgtgggtctcgcggtatcattgcagcactggggccagatggtaagccctcccgtatcgtagttatctacacgacggggagtcaggcaactatggatgaacgaaatagacagatcgctgagataggtgcctcactgattaagcattggtaactgtcagaccaagtttactcatatatactttagattgatttctgaaagcgaccaggtgctcggcgtggcaagactcgcagcgaacccgtagaaagccatgctccagccgcccgcattggagaaattcttcaaattcccgttgcacatagcccggcaattcctttccctgctctgccataagcgcagcgaatgccgggtaatactcgtcaacgatctgatagagaagggtttgctcgggtcggtggctctggtaacgaccagtatcccgatcccggctggccgtcctggccgccacatgaggcatgttccgcgtccttgcaatactgtgtttacatacagtctatcgcttagcggaaagttcttttaccctcagccgaaatgcctgccgttgctagacattgccagccagtgcccgtcactcccgtactaactgtcacgaacccctgcaataactgtcacgcccccctgcaataactgtcacgaacccctgcaataactgtcacgcccccaaacctgcaaacccagcaggggcgggggctggcggggtgttggaaaaatccatccatgattatctaagaataatccactaggcgcggttatcagcgcccttgtggggcgctgctgcccttgcccaatatgcccggccagaggccggatagctggtctattcgctgcgctaggctacacaccgccccaccgctgcgcggcagggggaaaggcgggcaaagcccgctaaaccccacaccaaaccccgcagaaatacgctggagcgcttttagccgctttagcggcctttccccctacccgaagggtgggggcgcgtgtgcagccccgcagggcctgtctcggtcgatcattcagcccggctcatccttctggcgtggcggcagaccgaacaaggcgcggtcgtggtcgcgttcaaggtacgcatccattgccgccatgagccgatcctccggccactcgctgctgttcaccttggccaaaatcatggcccccaccagcaccttgcgccttgtttcgttcttgcgctcttgctgctgttcccttgcccgctcccgctgaatttcggcattgattcgcgctcgttgttcttcgagcttggccagccgatccgccgccttgttgctccccttaaccatcttgacaccccattgttaatgtgctgtctcgtaggctatcatggaggcacagcggcggcaatcccgaccctactttgtaggggagggcgcacttaccggtttctcttcgagaaactggcctaacggccacccttcgggcggtgcgctctccgagggccattgcatggagccgaaaagcaaaagcaacagcgaggcagcatggcgatttatcaccttacggcgaaaaccggcagcaggtcgggcggccaatcggccagggccaaggccgactacatccagcgcgaaggcaagtatgcccgcgacatggatgaagtcttgcacgccgaatccgggcacatgccggagttcgtcgagcggcccgccgactactgggatgctgccgacctgtatgaacgcgccaatgggcggctgttcaaggaggtcgaatttgccctgccggtcgagctgaccctcgaccagcagaaggcgctggcgtccgagttcgcccagcacctgaccggtgccgagcgcctgccgtatacgctggccatccatgccggtggcggcgagaacccgcactgccacctgatgatctccgagcggatcaatgacggcatcgagcggcccgccgctcagtggttcaagcggtacaacggcaagaccccggagaagggcggggcacagaagaccgaagcgctcaagcccaaggcatggcttgagcagacccgcgaggcatgggccgaccatgccaaccgggcattagagcgggctggccacgacgcccgcattgaccacagaacacttgaggcgcagggcatcgagcgcctgcccggtgttcacctggggccgaacgtggtggagatggaaggccggggcatccgcaccgaccgggcagacgtggccctgaacatcgacaccgccaacgcccagatcatcgacttacaggaataccgggaggcaatagaccatgaacgcaatcgacagagtgaagaaatccagaggcatcaacgagttagcggagcagatcgaaccgctggcccagagcatggcgacactggccgacgaagcccggcaggtcatgagccagaccaagcaggccagcgaggcgcaggcggcggagtggctgaaagcccagcgccagacaggggcggcatgggtggagctggccaaagagttgcgggaggtagccgccgaggtgagcagcgccgcgcagagcgcccggagcgcgtcgcgggggtggcactggaagctatggctaaccgtgatgctggcttccatgatgcctacggtggtgctgctgatcgcatcgttgctcttgctcgacctgacgccactgacaaccgaggacggctcgatctggctgcgcttggtggcccgatgaagaacgacaggactttgcaggccataggccgacagctcaaggccatgggctgtgagcgcttcgatatcggcgtcagggacgcacccaccggccagatgatgaaccgggaatggtcagccgccgaagtgctccagaacacgccatggctcaagcggatgaatgcccagggcaatgacgtgtatatcaggcccgccgagcaggagcggcatggtctggtgctggtggacgacctcagcgagtttgacctggatgacatgaaagccgagggccgggagcctgccctggtagtggaaaccagcccgaagaactatcaggcatgggtcaaggtggccgacgccgcaggcggtgaacttcgggggcagattgcccggacgctggccagcgagtacgacgccgacccggccagcgccgacagccgccactatggccgcttggcgggcttcaccaaccgcaaggacaagcacaccacccgcgccggttatcagccgtgggtgctgctgcgtgaatccaagggcaagaccgccaccgctggcccggcgctggtgcagcaggctggccagcagatcgagcaggcccagcggcagcaggagaaggcccgcaggctggccagcctcgaactgcccgagcggcagcttagccgccaccggcgcacggcgctggacgagtaccgcagcgagatggccgggctggtcaagcgcttcggtcatgacctcagcaagtgcgactttatcgccgcgcagaagctggccagccggggccgcagtgccgaggaaatcggcaaggccatggccgaggccagcccagcgctggcagagcgcaagcccggccacgaagcggattacatcgagcgcaccgtcagcaaggtcatgggtctgcccagcgtccagcttgcgcgggccgagctggcacgggcaccggcaccccgccagcgaggcatggacaggggcgggccagatttcagcatgtagtgcttgcgttggtactcacgcctgttatactatgagtactcacgcacagaagggggttttatggaatacgaaaaaagcgcttcagggtcggtctacctgatcaaaagtgacaagggctattggttgcccggtggctttggttatacgtcaaacaaggccgaggctggccgcttttcagtcgctgatatggccagccttaaccttgacggctgcaccttgtccttgttccgcgaagacaagcctttcggccccggcaagtttctcggtgactgatatgaaagaccaaaaggacaagcagaccggcgacctgctggccagccctgacgctgtacgccaagcgcgatatgccgagcgcatgaaggccaaagggatgcgtcagcgcaagttctggctgaccgacgacgaatacgaggcgctgcgcgagtgcctggaagaactcagagcggcgcagggcgggggtagtgaccccgccagcgcctaaccaccaactgcctgcaaaggaggcaatcaatggctacccataagcctatcaatattctggaggcgttcgcagcagcgccgccaccgctggactacgttttgcccaacatggtggccggtacggtcggggcgctggtgtcgcccggtggtgccggtaaatccatgctggccctgcaactggccgcacagattgcaggcgggccggatctgctggaggtgggcgaactgcccaccggcccggtgatctacctgcccgccgaagacccgcccaccgccattcatcaccgcctgcacgcccttggggcgcacctcagcgccgaggaacggcaagccgtggctgacggcctgctgatccagccgctgatcggcagcctgcccaacatcatggccccggagtggttcgacggcctcaagcgcgccgccgagggccgccgcctgatggtgctggacacgctgcgccggttccacatcgaggaagaaaacgccagcggccccatggcccaggtcatcggtcgcatggaggccatcgccgccgataccgggtgctctatcgtgttcctgcaccatgccagcaagggcgcggccatgatgggcgcaggcgaccagcagcaggccagccggggcagctcggtactggtcgataacatccgctggcagtcctacctgtcgagcatgaccagcgccgaggccgaggaatggggtgtggacgacgaccagcgccggttcttcgtccgcttcggtgtgagcaaggccaactatggcgcaccgttcgctgatcggtggttcaggcggcatgacggcggggtgctcaagcccgccgtgctggagaggcagcgcaagagcaagggggtgccccgtggtgaagcctaagaacaagcacagcctcagccacgtccggcacgacccggcgcactgtctggcccccggcctgttccgtgccctcaagcggggcgagcgcaagcgcagcaagctggacgtgacgtatgactacggcgacggcaagcggatcgagttcagcggcccggagccgctgggcgctgatgatctgcgcatcctgcaagggctggtggccatggctgggcctaatggcctagtgcttggcccggaacccaagaccgaaggcggacggcagctccggctgttcctggaacccaagtgggaggccgtcaccgctgaatgccatgtggtcaaaggtagctatcgggcgctggcaaaggaaatcggggcagaggtcgatagtggtggggcgctcaagcacatacaggactgcatcgagcgcctttggaaggtatccatcatcgcccagaatggccgcaagcggcaggggtttcggctgctgtcggagtacgccagcgacgaggcggacgggcgcctgtacgtggccctgaaccccttgatcgcgcaggccgtcatgggtggcggccagcatgtgcgcatcagcatggacgaggtgcgggcgctggacagcgaaaccgcccgcctgctgcaccagcggctgtgtggctggatcgaccccggcaaaaccggcaaggcttccatagataccttgtgcggctatgtctggccgtcagaggccagtggttcgaccatgcgcaagcgccgcaagcgggtgcgcgaggcgttgccggagctggtcgcgctgggctggacggtaaccgagttcgcggcgggcaagtacgacatcacccggcccaaggcggcaggctgaccccccccactctattgtaaacaagacatttttatcttttatattcaatggcttattttcctgctaattggtaataccatgaaaaataccatgctcagaaaaggcttaacaatattttgaaaaattgcctactgagcgctgccgcacagctccataggccgctttcctggctttgcttccagatgtatgctcttctgctcccgaacgccagcaagacgtagcccagcgcgtcggccagcttgcaattcgcgctaacttacattaattgcgttgcgctcactgcccgctttccagtcgggaaacctgtcgtgccagctgcattaatgaatcggccaacgcgcggggagaggcggtttgcgtattgggcgccagggtggtttttcttttcaccagtgagacgggcaacagctgattgcccttcaccgcctggccctgagagagttgcagcaagcggtccacgtggtttgccccagcaggcgaaaatcctgtttgatggtggttaacggcgggatataacatgagctgtcttcggtatcgtcgtatcccactaccgagatatccgcaccaacgcgcagcccggactcggtaatggcgcgcattgcgcccagcgccatctgatcgttggcaaccagcatcgcagtgggaacgatgccctcattcagcatttgcatggtttgttgaaaaccggacatggcactccagtcgccttcccgttccgctatcggctgaatttgattgcgagtgagatatttatgccagccagccagacgcagacgcgccgagacagaacttaatgggcccgctaacagcgcgatttgctggtgacccaatgcgaccagatgctccacgcccagtcgcgtaccgtcttcatgggagaaaataatactgttgatgggtgtctggtcagagacatcaagaaataacgccggaacattagtgcaggcagcttccacagcaatggcatcctggtcatccagcggatagttaatgatcagcccactgacgcgttgccgatacaagaacaacagcgcgttgagcgcctgccggtgggtggccggcgccacttgcttctcggtggcgagcatggtcagaaaaccctcgacttcagcttgccccatttcgcgcggatgtcgaaacccaccatggctgcgggccgtccacaacacaaatgccttggcccagtagacataagccttctcggtctgtaggctgtaatgcaggtagcgaacccgtgaa

2. pMMPc-mCherry2 ttcaaagaaggagatatacatATGGTGTCGAAGGGCGAAGAAGACAACATGGCCATCATCAAGGAATTCATGCGCTTCAAGGTGCACATGGAAGGCTCGGTGAACGGCCACGAATTCGAAATCGAAGGCGAAGGCGAAGGCCGCCCGTACGAAGGCACCCAGACCGCCAAGCTGAAGGTGACCAAGGGCGGCCCGCTGCCGTTCGCCTGGGACATCCTGTCGCCGCAGTTCATGTACGGCTCGAAGGCCTACGTGAAGCACCCGGCCGACATCCCGGACTACCTGAAGCTGTCGTTCCCGGAAGGCTTCAAGTGGGAACGCGTGATGAACTTCGAAGACGGCGGCGTGGTGACCGTGACCCAGGACTCGTCGCTGCAGGACGGCGAATTCATCTACAAGGTGAAGCTGCGCGGCACCAACTTCCCGTCGGACGGCCCGGTGATGCAGAAGAAGACCATGGGCTGGGAAGCCTCGTCGGAACGCATGTACCCGGAAGACGGCGCCCTGAAGGGCGAAATCAAGCAGCGCCTGAAGCTGAAGGACGGCGGCCACTACGACGCCGAAGTGAAGACCACCTACAAGGCCAAGAAGCCGGTGCAGCTGCCGGGCGCCTACAACGTGAACATCAAGCTGGACATCACCTCGCACAACGAAGACTACACCATCGTGGAACAGTACGAACGCGCCGAAGGCCGCCACTCGACCGGCGGCATGGACGAACTGTACAAGTAAaagcttttaggtggcggtacttgggtcgatatcaaagtgcatcacttcttcccgtatgcccaactttgtatagagagccactgcgggatcgtcaccgtaatctgcttgcacgtagatcacataagcaccaagcgcgttggcctcatgcttgaggagattgatgagcgcggtggcaatgccctgcctccggtgctcgccggagactgcgagatcatagatatagatctcactacgcggctgctcaaacttgggcagaacgtaagccgcgagagcgccaacaaccgcttcttggtcgaaggcagcaagcgcgatgaatgtcttactacggagcaagttcccgaggtaatcggagtccggctgatgttgggagtaggtggctacgtctccgaactcacgaccgaaaagatcaagagcagcccgcatggatttgacttggtcagggccgagcctacatgtgcgaatgatgcccatacttgagccacctaactttgttttagggcgactgccctgctgcgtaacatcgttgctgctgcgtaacatcgttgctgctccataacatcaaacatcgacccacggcgtaacgcgcttgctgcttggatgcccgaggcatagactgtacaaaaaaacagtcataacaagccatgaaaaccgccactgcgccgttaccaccgctgcgttcggtcaaggttctggaccagttgcgtgagcgcatacgctacttgcattacagtttacgaaccgaacaggcttatgtcaattcgatccattgctgttgacaaagggaatcagcgatcggaggaccgaaggagctaaccgcttttttgcacaacatgggggatcatgtaactcgccttgatcgttgggaaccggagctgaatgaagccataccaaacgacgagcgtgacaccacgatgcctgtagcaatggcaacaacgttgcgcaaactattaactggcgaactacttactctagcttcccggcaacaattaatagactggatggaggcggataaagttgcaggaccacttctgcgctcggcccttccggctggctggtttattgctgataaatctggagccggtgagcgtgggtctcgcggtatcattgcagcactggggccagatggtaagccctcccgtatcgtagttatctacacgacggggagtcaggcaactatggatgaacgaaatagacagatcgctgagataggtgcctcactgattaagcattggtaactgtcagaccaagtttactcatatatactttagattgatttctgaaagcgaccaggtgctcggcgtggcaagactcgcagcgaacccgtagaaagccatgctccagccgcccgcattggagaaattcttcaaattcccgttgcacatagcccggcaattcctttccctgctctgccataagcgcagcgaatgccgggtaatactcgtcaacgatctgatagagaagggtttgctcgggtcggtggctctggtaacgaccagtatcccgatcccggctggccgtcctggccgccacatgaggcatgttccgcgtccttgcaatactgtgtttacatacagtctatcgcttagcggaaagttcttttaccctcagccgaaatgcctgccgttgctagacattgccagccagtgcccgtcactcccgtactaactgtcacgaacccctgcaataactgtcacgcccccctgcaataactgtcacgaacccctgcaataactgtcacgcccccaaacctgcaaacccagcaggggcgggggctggcggggtgttggaaaaatccatccatgattatctaagaataatccactaggcgcggttatcagcgcccttgtggggcgctgctgcccttgcccaatatgcccggccagaggccggatagctggtctattcgctgcgctaggctacacaccgccccaccgctgcgcggcagggggaaaggcgggcaaagcccgctaaaccccacaccaaaccccgcagaaatacgctggagcgcttttagccgctttagcggcctttccccctacccgaagggtgggggcgcgtgtgcagccccgcagggcctgtctcggtcgatcattcagcccggctcatccttctggcgtggcggcagaccgaacaaggcgcggtcgtggtcgcgttcaaggtacgcatccattgccgccatgagccgatcctccggccactcgctgctgttcaccttggccaaaatcatggcccccaccagcaccttgcgccttgtttcgttcttgcgctcttgctgctgttcccttgcccgctcccgctgaatttcggcattgattcgcgctcgttgttcttcgagcttggccagccgatccgccgccttgttgctccccttaaccatcttgacaccccattgttaatgtgctgtctcgtaggctatcatggaggcacagcggcggcaatcccgaccctactttgtaggggagggcgcacttaccggtttctcttcgagaaactggcctaacggccacccttcgggcggtgcgctctccgagggccattgcatggagccgaaaagcaaaagcaacagcgaggcagcatggcgatttatcaccttacggcgaaaaccggcagcaggtcgggcggccaatcggccagggccaaggccgactacatccagcgcgaaggcaagtatgcccgcgacatggatgaagtcttgcacgccgaatccgggcacatgccggagttcgtcgagcggcccgccgactactgggatgctgccgacctgtatgaacgcgccaatgggcggctgttcaaggaggtcgaatttgccctgccggtcgagctgaccctcgaccagcagaaggcgctggcgtccgagttcgcccagcacctgaccggtgccgagcgcctgccgtatacgctggccatccatgccggtggcggcgagaacccgcactgccacctgatgatctccgagcggatcaatgacggcatcgagcggcccgccgctcagtggttcaagcggtacaacggcaagaccccggagaagggcggggcacagaagaccgaagcgctcaagcccaaggcatggcttgagcagacccgcgaggcatgggccgaccatgccaaccgggcattagagcgggctggccacgacgcccgcattgaccacagaacacttgaggcgcagggcatcgagcgcctgcccggtgttcacctggggccgaacgtggtggagatggaaggccggggcatccgcaccgaccgggcagacgtggccctgaacatcgacaccgccaacgcccagatcatcgacttacaggaataccgggaggcaatagaccatgaacgcaatcgacagagtgaagaaatccagaggcatcaacgagttagcggagcagatcgaaccgctggcccagagcatggcgacactggccgacgaagcccggcaggtcatgagccagaccaagcaggccagcgaggcgcaggcggcggagtggctgaaagcccagcgccagacaggggcggcatgggtggagctggccaaagagttgcgggaggtagccgccgaggtgagcagcgccgcgcagagcgcccggagcgcgtcgcgggggtggcactggaagctatggctaaccgtgatgctggcttccatgatgcctacggtggtgctgctgatcgcatcgttgctcttgctcgacctgacgccactgacaaccgaggacggctcgatctggctgcgcttggtggcccgatgaagaacgacaggactttgcaggccataggccgacagctcaaggccatgggctgtgagcgcttcgatatcggcgtcagggacgcacccaccggccagatgatgaaccgggaatggtcagccgccgaagtgctccagaacacgccatggctcaagcggatgaatgcccagggcaatgacgtgtatatcaggcccgccgagcaggagcggcatggtctggtgctggtggacgacctcagcgagtttgacctggatgacatgaaagccgagggccgggagcctgccctggtagtggaaaccagcccgaagaactatcaggcatgggtcaaggtggccgacgccgcaggcggtgaacttcgggggcagattgcccggacgctggccagcgagtacgacgccgacccggccagcgccgacagccgccactatggccgcttggcgggcttcaccaaccgcaaggacaagcacaccacccgcgccggttatcagccgtgggtgctgctgcgtgaatccaagggcaagaccgccaccgctggcccggcgctggtgcagcaggctggccagcagatcgagcaggcccagcggcagcaggagaaggcccgcaggctggccagcctcgaactgcccgagcggcagcttagccgccaccggcgcacggcgctggacgagtaccgcagcgagatggccgggctggtcaagcgcttcggtcatgacctcagcaagtgcgactttatcgccgcgcagaagctggccagccggggccgcagtgccgaggaaatcggcaaggccatggccgaggccagcccagcgctggcagagcgcaagcccggccacgaagcggattacatcgagcgcaccgtcagcaaggtcatgggtctgcccagcgtccagcttgcgcgggccgagctggcacgggcaccggcaccccgccagcgaggcatggacaggggcgggccagatttcagcatgtagtgcttgcgttggtactcacgcctgttatactatgagtactcacgcacagaagggggttttatggaatacgaaaaaagcgcttcagggtcggtctacctgatcaaaagtgacaagggctattggttgcccggtggctttggttatacgtcaaacaaggccgaggctggccgcttttcagtcgctgatatggccagccttaaccttgacggctgcaccttgtccttgttccgcgaagacaagcctttcggccccggcaagtttctcggtgactgatatgaaagaccaaaaggacaagcagaccggcgacctgctggccagccctgacgctgtacgccaagcgcgatatgccgagcgcatgaaggccaaagggatgcgtcagcgcaagttctggctgaccgacgacgaatacgaggcgctgcgcgagtgcctggaagaactcagagcggcgcagggcgggggtagtgaccccgccagcgcctaaccaccaactgcctgcaaaggaggcaatcaatggctacccataagcctatcaatattctggaggcgttcgcagcagcgccgccaccgctggactacgttttgcccaacatggtggccggtacggtcggggcgctggtgtcgcccggtggtgccggtaaatccatgctggccctgcaactggccgcacagattgcaggcgggccggatctgctggaggtgggcgaactgcccaccggcccggtgatctacctgcccgccgaagacccgcccaccgccattcatcaccgcctgcacgcccttggggcgcacctcagcgccgaggaacggcaagccgtggctgacggcctgctgatccagccgctgatcggcagcctgcccaacatcatggccccggagtggttcgacggcctcaagcgcgccgccgagggccgccgcctgatggtgctggacacgctgcgccggttccacatcgaggaagaaaacgccagcggccccatggcccaggtcatcggtcgcatggaggccatcgccgccgataccgggtgctctatcgtgttcctgcaccatgccagcaagggcgcggccatgatgggcgcaggcgaccagcagcaggccagccggggcagctcggtactggtcgataacatccgctggcagtcctacctgtcgagcatgaccagcgccgaggccgaggaatggggtgtggacgacgaccagcgccggttcttcgtccgcttcggtgtgagcaaggccaactatggcgcaccgttcgctgatcggtggttcaggcggcatgacggcggggtgctcaagcccgccgtgctggagaggcagcgcaagagcaagggggtgccccgtggtgaagcctaagaacaagcacagcctcagccacgtccggcacgacccggcgcactgtctggcccccggcctgttccgtgccctcaagcggggcgagcgcaagcgcagcaagctggacgtgacgtatgactacggcgacggcaagcggatcgagttcagcggcccggagccgctgggcgctgatgatctgcgcatcctgcaagggctggtggccatggctgggcctaatggcctagtgcttggcccggaacccaagaccgaaggcggacggcagctccggctgttcctggaacccaagtgggaggccgtcaccgctgaatgccatgtggtcaaaggtagctatcgggcgctggcaaaggaaatcggggcagaggtcgatagtggtggggcgctcaagcacatacaggactgcatcgagcgcctttggaaggtatccatcatcgcccagaatggccgcaagcggcaggggtttcggctgctgtcggagtacgccagcgacgaggcggacgggcgcctgtacgtggccctgaaccccttgatcgcgcaggccgtcatgggtggcggccagcatgtgcgcatcagcatggacgaggtgcgggcgctggacagcgaaaccgcccgcctgctgcaccagcggctgtgtggctggatcgaccccggcaaaaccggcaaggcttccatagataccttgtgcggctatgtctggccgtcagaggccagtggttcgaccatgcgcaagcgccgcaagcgggtgcgcgaggcgttgccggagctggtcgcgctgggctggacggtaaccgagttcgcggcgggcaagtacgacatcacccggcccaaggcggcaggctgaccccccccactctattgtaaacaagacatttttatcttttatattcaatggcttattttcctgctaattggtaataccatgaaaaataccatgctcagaaaaggcttaacaatattttgaaaaattgcctactgagcgctgccgcacagctccataggccgctttcctggctttgcttccagatgtatgctcttctgctcccgaacgccagcaagacgtagcccagcgcgtcggccagcttgcaattcgcgctaacttacattaattgcgttgcgctcactgcccgctttccagtcgggaaacctgtcgtgccagctgcattaatgaatcggccaacgcgcggggagaggcggtttgcgtattgggcgccagggtggtttttcttttcaccagtgagacgggcaacagctgattgcccttcaccgcctggccctgagagagttgcagcaagcggtccacgtggtttgccccagcaggcgaaaatcctgtttgatggtggttaacggcgggatataacatgagctgtcttcggtatcgtcgtatcccactaccgagatatccgcaccaacgcgcagcccggactcggtaatggcgcgcattgcgcccagcgccatctgatcgttggcaaccagcatcgcagtgggaacgatgccctcattcagcatttgcatggtttgttgaaaaccggacatggcactccagtcgccttcccgttccgctatcggctgaatttgattgcgagtgagatatttatgccagccagccagacgcagacgcgccgagacagaacttaatgggcccgctaacagcgcgatttgctggtgacccaatgcgaccagatgctccacgcccagtcgcgtaccgtcttcatgggagaaaataatactgttgatgggtgtctggtcagagacatcaagaaataacgccggaacattagtgcaggcagcttccacagcaatggcatcctggtcatccagcggatagttaatgatcagcccactgacgcgttgccgatacaagaacaacagcgcgttgagcgcctgccggtgggtggccggcgccacttgcttctcggtggcgagcatggtcagaaaaccctcgacttcagcttgccccatttcgcgcggatgtcgaaacccaccatggctgcgggccgtccacaacacaaatgccttggcccagtagacataagccttctcggtctgtaggctgtaatgcaggtagcgaacccgtgaa

Table 3 Primers used in this study

| Gene | Primer name | Primer sequence (5’-3’) | Application |
| --- | --- | --- | --- |
| *egfp2* | egfp-pMMpc-F | tcaaagaaggagatatacatATGGTGTCGAAGGGCGAAGAACT | Amplication of *egfp2* |
|  | egfp-pMMpc-R | gtaccgccacctaaaagcttTTACTTGTACAGTTCGTCCATGCCCAGG |  |
| *mcherry2* | mcherry-pMMpc-F | tcaaagaaggagatatacatATGGTGTCGAAGGGC | Amplication of *mcherry2* |
|  | mcherry-pMMpc-R | gtaccgccacctaaaagcttTTACTTGTACAG |  |
| pMMPc fragment | pMMpc-kuozeng-F | aagcttttaggtggcggtacttggg | Amplication of pMMPc fragment |
|  | pMMpc-kuozeng-R | atgtatatctccttctttgaattcacgggttcgctac |  |
| pMMPc-egfp2 and pMMPc-mcherry2 | pMMpc-F | atttgctggtgacccaatgcg | pMMPc-egfp2 and pMMPc-mcherry2 construction |
|  | pMMpc-R | gtaggctcggccctgac |  |

# **S2 Supplementary Tables**

Table 4 Definitions of variables in the IBM

| Variable | Description | Units |
| --- | --- | --- |
| $S_{1}$ | The concentration of substrate in the detoxifier | C-mmol |
| $S_{2}$ | The concentration of substrate in the consumer | C-mmol |
| $I_{1}$ | The concentration of intermediate in the detoxifier | C-mmol |
| $I_{2}$ | The concentration of intermediate in the consumer | C-mmol |
| $P_{1}$ | The concentration of the final product in the detoxifier | C-mmol |
| $P_{2}$ | The concentration of the final product in the consumer | C-mmol |
| $V_{1}$ | The volume of the detoxifier cells | fL |
| $V_{2}$ | The volume of the consumer cells | fL |
| $L_{1}$ | Length of the detoxifier cells | μm |
| $L_{2}$ | Length of the consumer cells | μm |

Table 5 Definition and value of parameters in IBM

| Parameter | Description | Default value and units | Source |
| --- | --- | --- | --- |
| $K_{1}$ | Michaelis-Menten constant first reaction. | 0.096 C-mmol⸱L^-1^ | Beatriz Ca´mara [9] |
| $E_{1}$ | Enzyme concentration of the first reaction. | 0.001-0.06 C-mmol | Steven B. Zimmerman [10] |
| $k_{1}$ | The specific rate of the first reaction. | 46 min^-1^ | Beatriz Ca´mara [9] |
| $K_{2}$ | Michaelis-Menten constant second reaction. | 0.015 C-mmol⸱L^-1^ | Ambra Viggiani [11] |
| $E_{2}$ | Enzyme concentration of the second reaction. | 0.02 C-mmol | Steven B. Zimmerman [10] |
| $k_{2}$ | The specific rate of the second reaction. | 2000 min^-1^ | Ambra Viggiani [11] |
| $D_{S}$ | Diffusivity coefficient for the substrate | 2.0 | The maximum value in *gro* |
| $D_{I}$ | Diffusivity coefficient for the intermediate | 2.0 | The maximum value in *gro* |
| $D_{P}$ | Diffusivity coefficient for the product | 0.005-2 | Miaoxiao Wang [7] |
| $Kg$ | The half-saturation constant of Monod growth. | 0.02 C-mmol⸱L^-1^ | Miaoxiao Wang [7] |
| $kg$ | The maximum consumption rate of the final product for cell growth. | 0.32 C-mmol⸱min^-1^ | Miaoxiao Wang[7] |
| $d$ | Apparent maintenance rate | 0.0003 min^-1^ | Miaoxiao Wang [7] |
| $w$ | Width (diameter) of the capsular cells | 1.0 μm | The default value in *gro* |
| $Y$ | Yield coefficient for biomass production | 0.1 C-mmol^-1^ | Miaoxiao Wang [7] |
| $C$ | Fitness cost of cells performing specific functions in the pathway | 1 | This study |
| $S$ | The initial concentration of the substrate | 10 C-mmol⸱L^-1^ |  |

# **S2 Supplementary Figures**


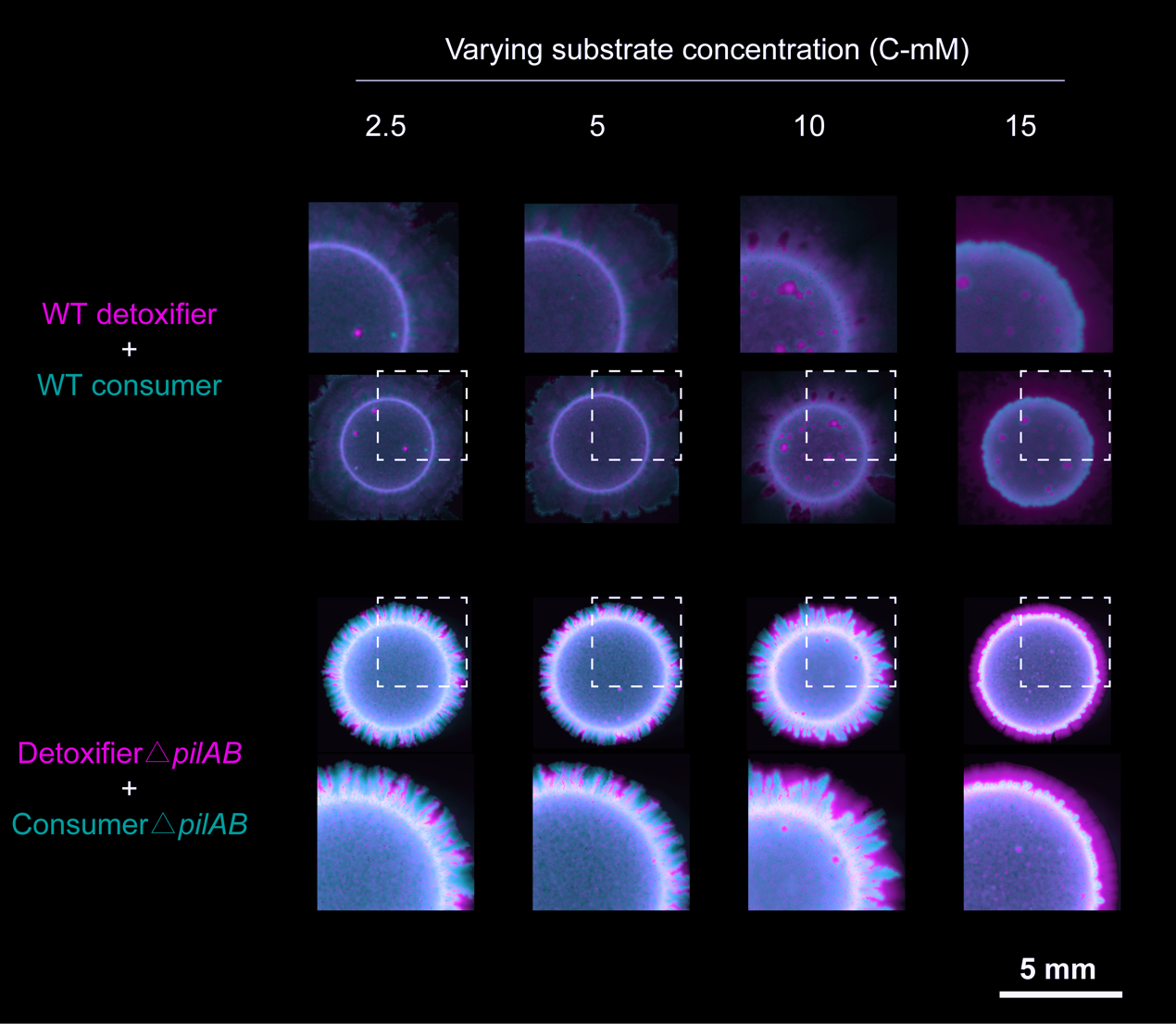


**Supplementary Figure 1** Expansion of microbial consortia composed of the wild-type detoxifier and consumer, or Δ*pilAB* detoxifier and consumer. Deletion of the *pilAB* gene in both the detoxifier and the consumer leads to the formation of well-defined spatial patterns. Therefore, all subsequent experiments were performed using Δ*pilAB* detoxifier and consumer strains.

**
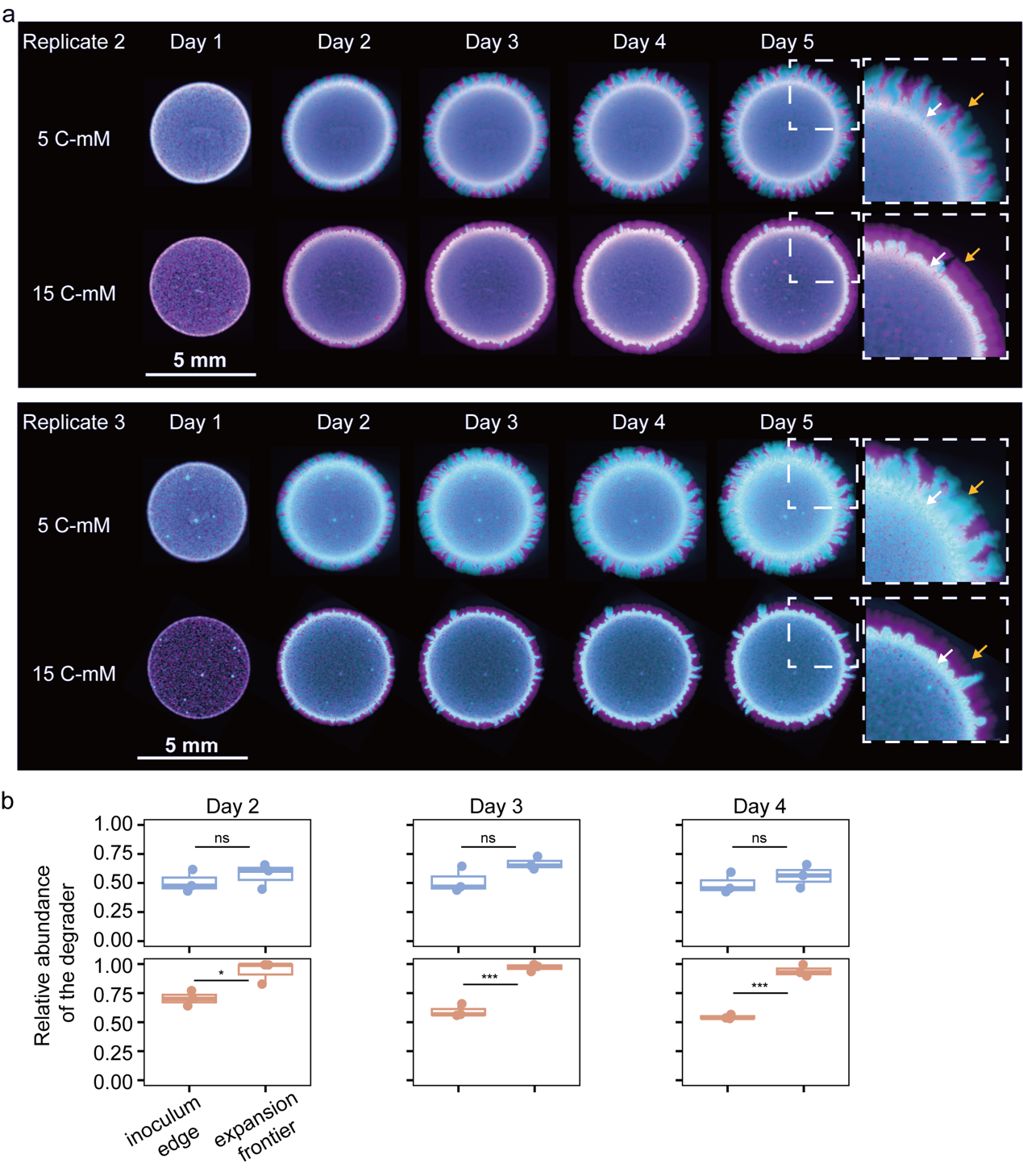
**

**Supplementary Figure 2** Effects of substrate concentration on the self-organization of microbial communities composed of the detoxifier and the consumer. (a) Two additional biological replicates of Fig. 1b and 1c are shown. The detoxifier and consumer were mixed and inoculated onto agar plates supplemented with salicylate as the growth substrate and incubated for five days. The detoxifier expressing the mCherry fluorescent protein is shown in magenta, while the consumer expressing the eGFP fluorescent protein is shown in cyan. Dashed boxes mark regions that are enlarged to highlight the inoculum edge and the expansion frontier. Inoculum edges are indicated by white arrows, whereas expansion frontiers are indicated by yellow arrows. (b) Relative abundance of the detoxifier at the inoculum edge and at the expansion frontier at different days after inoculation under two salicylate concentrations (5 and 15 C-mM). Under 15 C-mM salicylate, the detoxifier abundance at the expansion frontier was close to 1 and was significantly higher than that at the inoculum edge (n = 3). *P* values were derived by t-test and shown as ns: P > 0.05; *: 0.01 < *P* < 0.05; **: 0.001 < *P* < 0.01; ***: *P* < 0.001.


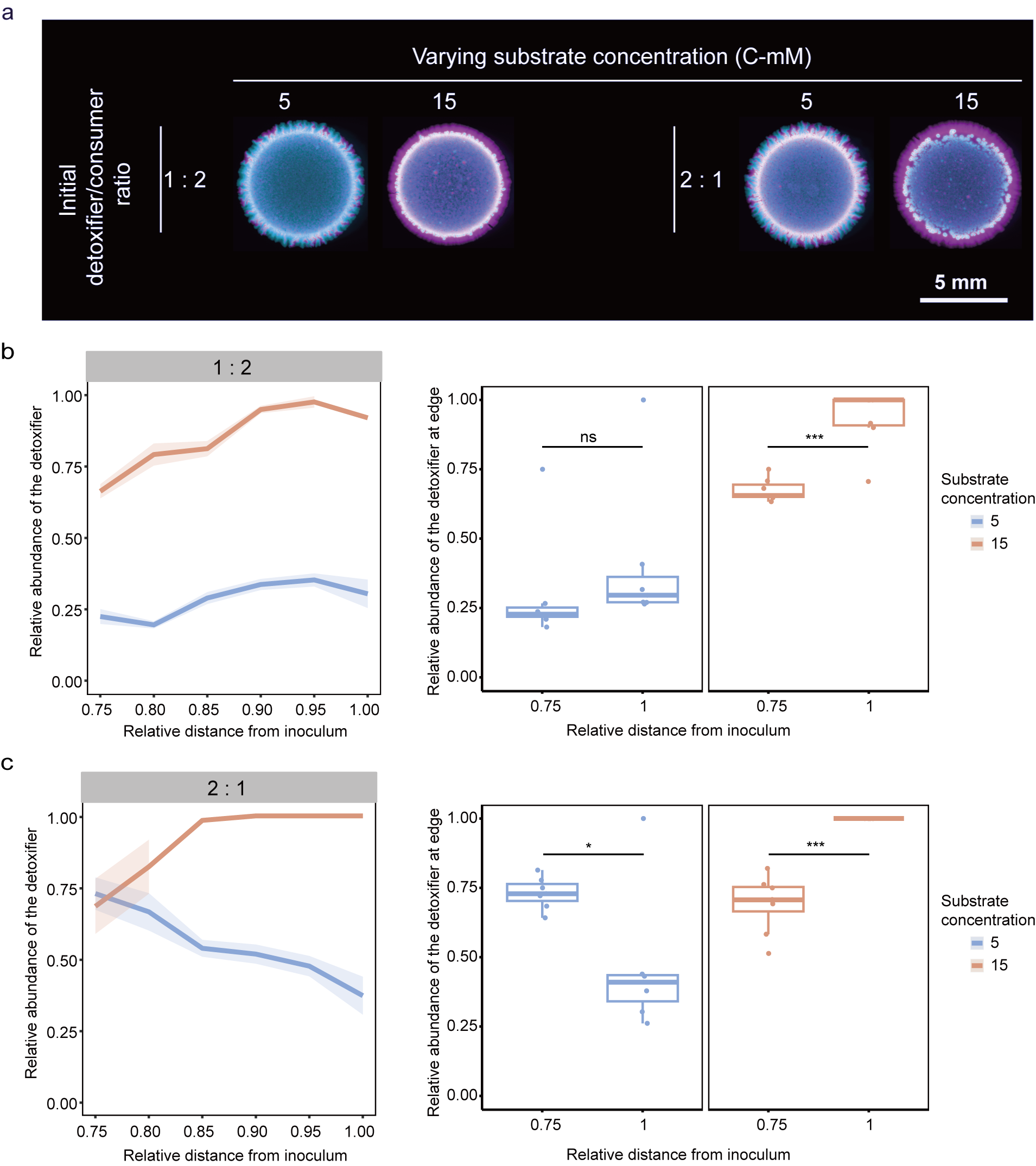


**Supplementary Figure 3** Spatial expansion colonies initiated at different ratios of the detoxifier cells to the consumer cells. (a) Colonies were grown with salicylate at a range of concentrations for five days and imaged by fluorescence microscopy. The detoxifier cells (magenta) were mixed with the consumer cells (cyan) at an initial ratio of 1:2 and 2:1, respectively. (b-c) Measurement of relative abundance of the detoxifier from the edge of the inoculum towards the edge of expansion (Left). Lines and shaded areas correspond to the mean of data points (n = 3) and 95% confidence interval of the mean, respectively. At a high salicylate concentration of 15 C-mM, the relative abundance of the detoxifier at the edge of the inoculum is significantly higher than at the edge of expansion frontier (Right). Two-tailed two-sample t test: *P* > 0.05 (ns); *P* < 0.05 (*); *P* < 0.01 (**); *P* < 0.001 (***).


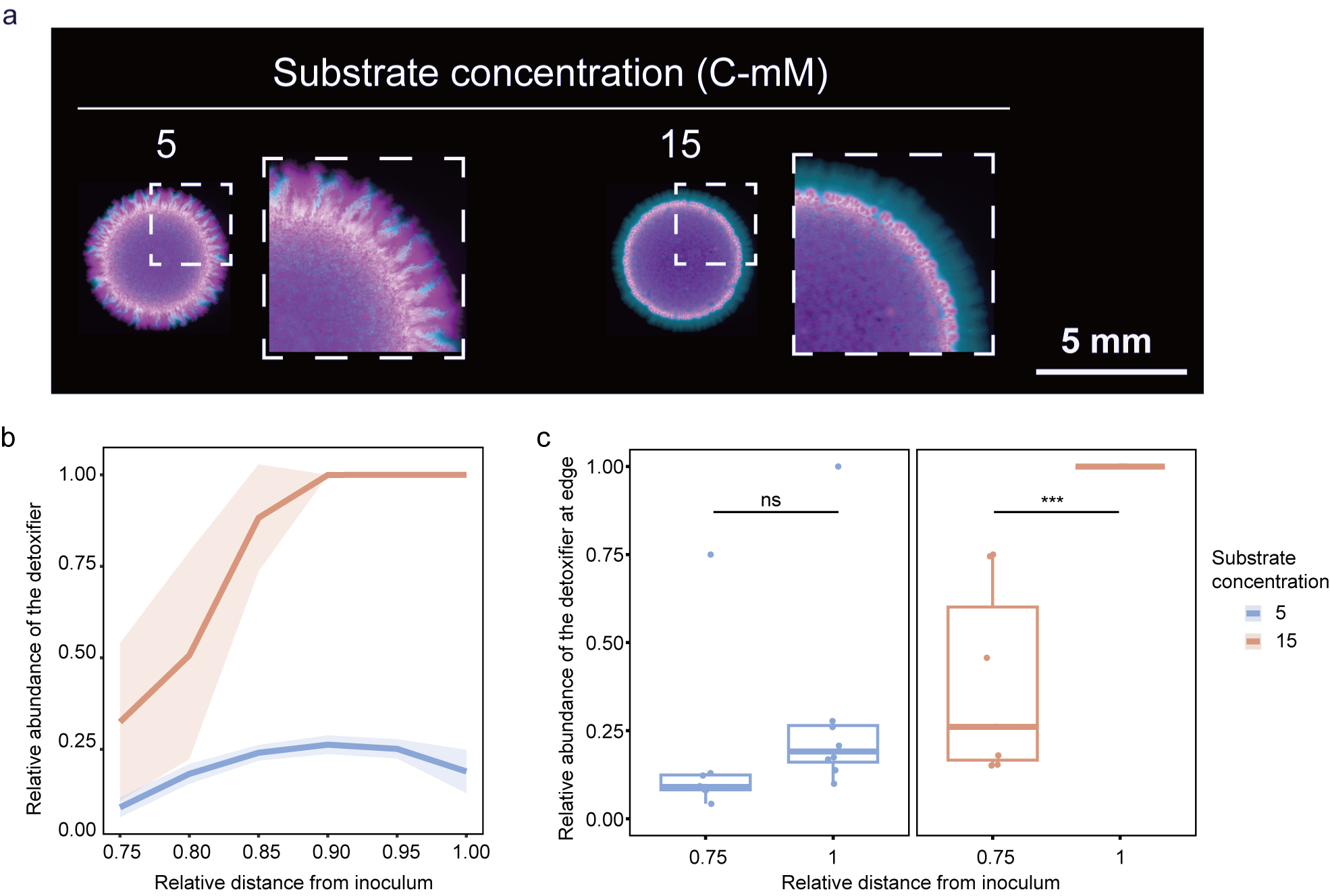


**Supplementary Figure 4** Spatial expansion colonies consisting of detoxifier cells (cyan) and the consumer cells (magenta). (a) Colonies were grown with salicylate at a range of concentrations for five days and imaged by fluorescence microscopy. (b) Measurement of relative abundance of the detoxifier from the edge of the inoculum towards the edge of expansion (Left). Lines and shaded areas correspond to the mean of data points (n = 3) and 95% confidence interval of the mean, respectively. Under the condition with 5 C-mM salicylate as the carbon source, the relative abundance of the detoxifier at the edge of expansion frontier is similar to that at the edge of the inoculum. However, under the condition with 15 C-mM salicylate as the carbon source, the relative abundance of the detoxifier at the edge of the inoculum is significantly higher than that at the edge of expansion frontier (Right). Two-tailed two-sample t test: *P* > 0.05 (ns); *P* < 0.05 (*); *P* < 0.01 (**); *P* < 0.001 (***).


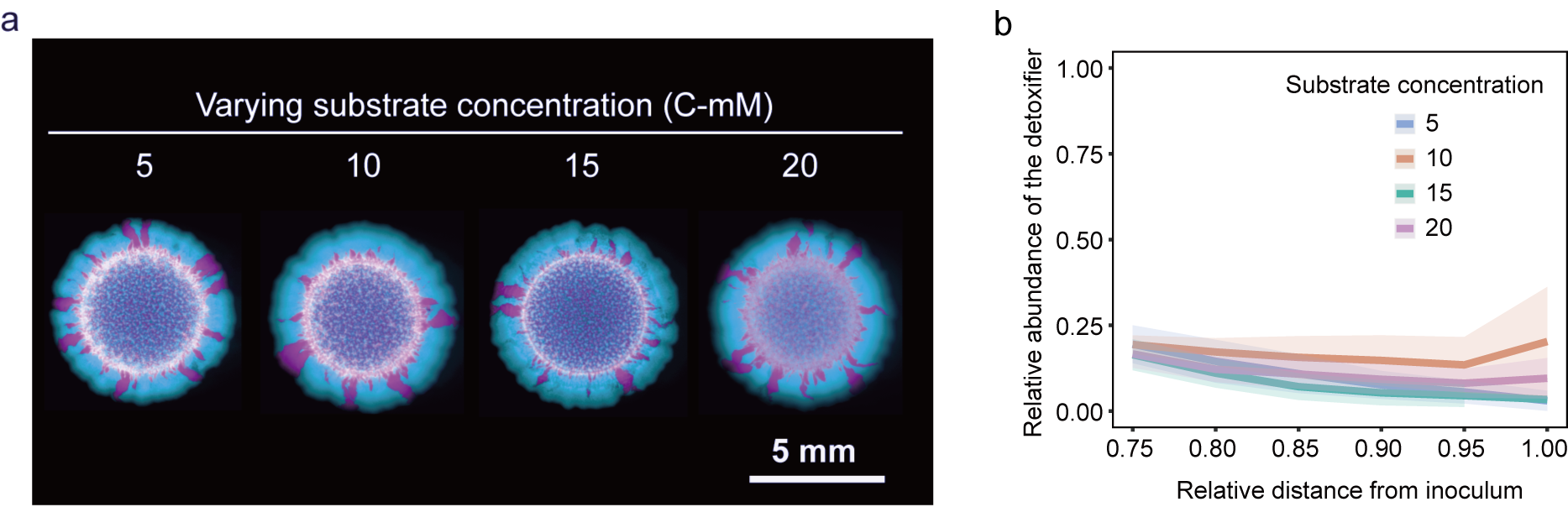


**Supplementary Figure 5** Effects of pyruvate concentrations on the self-organization of microbial communities consisting of the detoxifier and the consumer. (a) A representation of fluorescent stereomicroscope colony images with varying pyruvate concentrations. The detoxifier is colored magenta and the consumer is colored cyan. (b) The proportion of detoxifier cells at the colony periphery after five days of incubation. Shaded areas represent 95% confidence intervals (n = 3). We performed a one-sample t-test comparing the detoxifier fraction against 0.5 (null hypothesis: equal abundance of the two strains). The detoxifier fraction was significantly lower than 0.5 (*P* < 0.01).

**
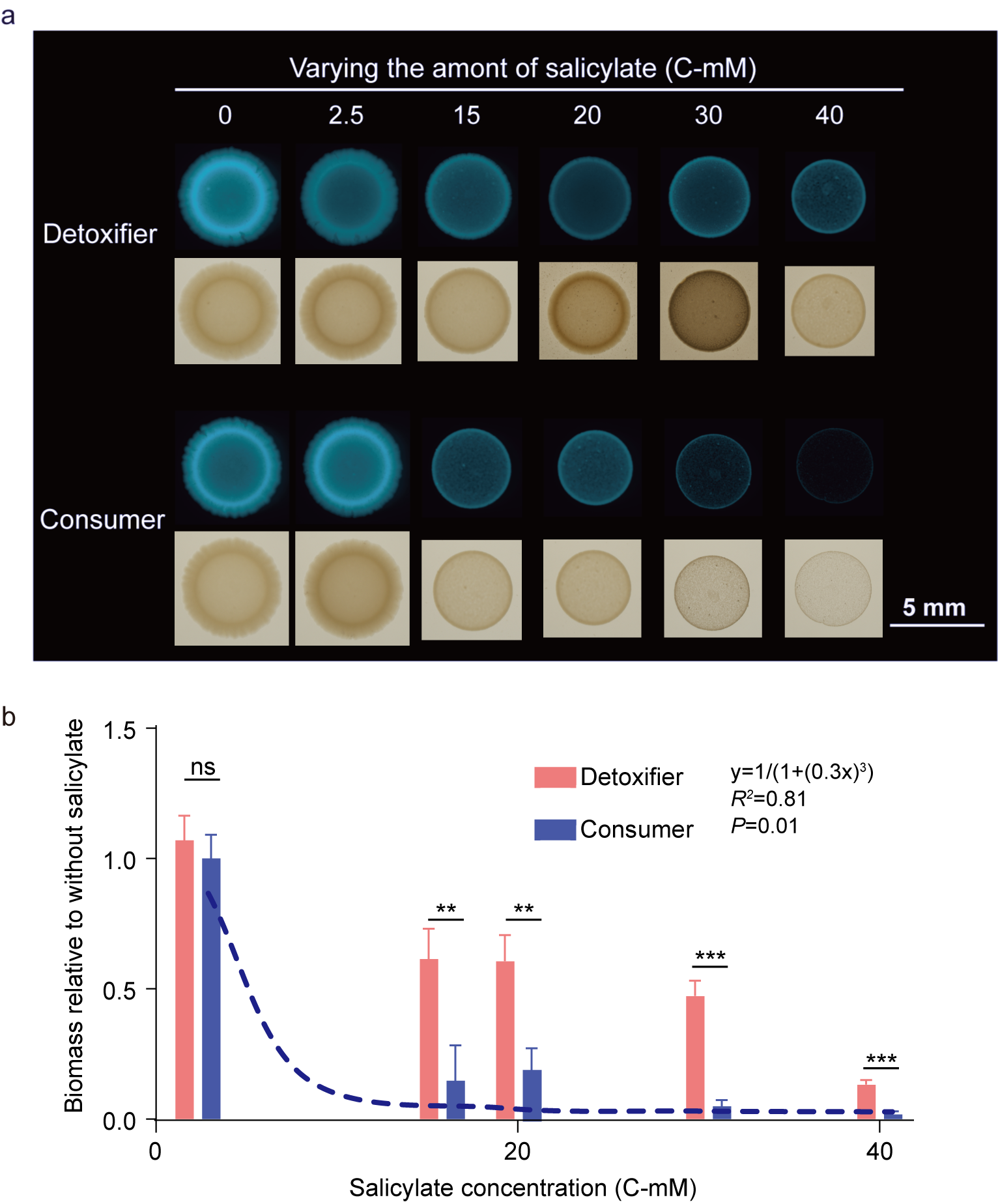
Supplementary Figure 6** Effects of salicylate concentrations on the growth yield of the detoxifier and consumer. The detoxifier displays much higher salicylate tolerance compared to the consumer. (a) A representation of stereomicroscope colony images with varying salicylate concentrations. (b) The ratio of the biomass of the strain when adding different concentrations of salicylate to the biomass of the strain without adding salicylate. Two-tailed two-sample *t* test: *P* > 0.05 (ns); *P* < 0.05 (*); *P* < 0.01 (**); *P* < 0.001 (***).

**
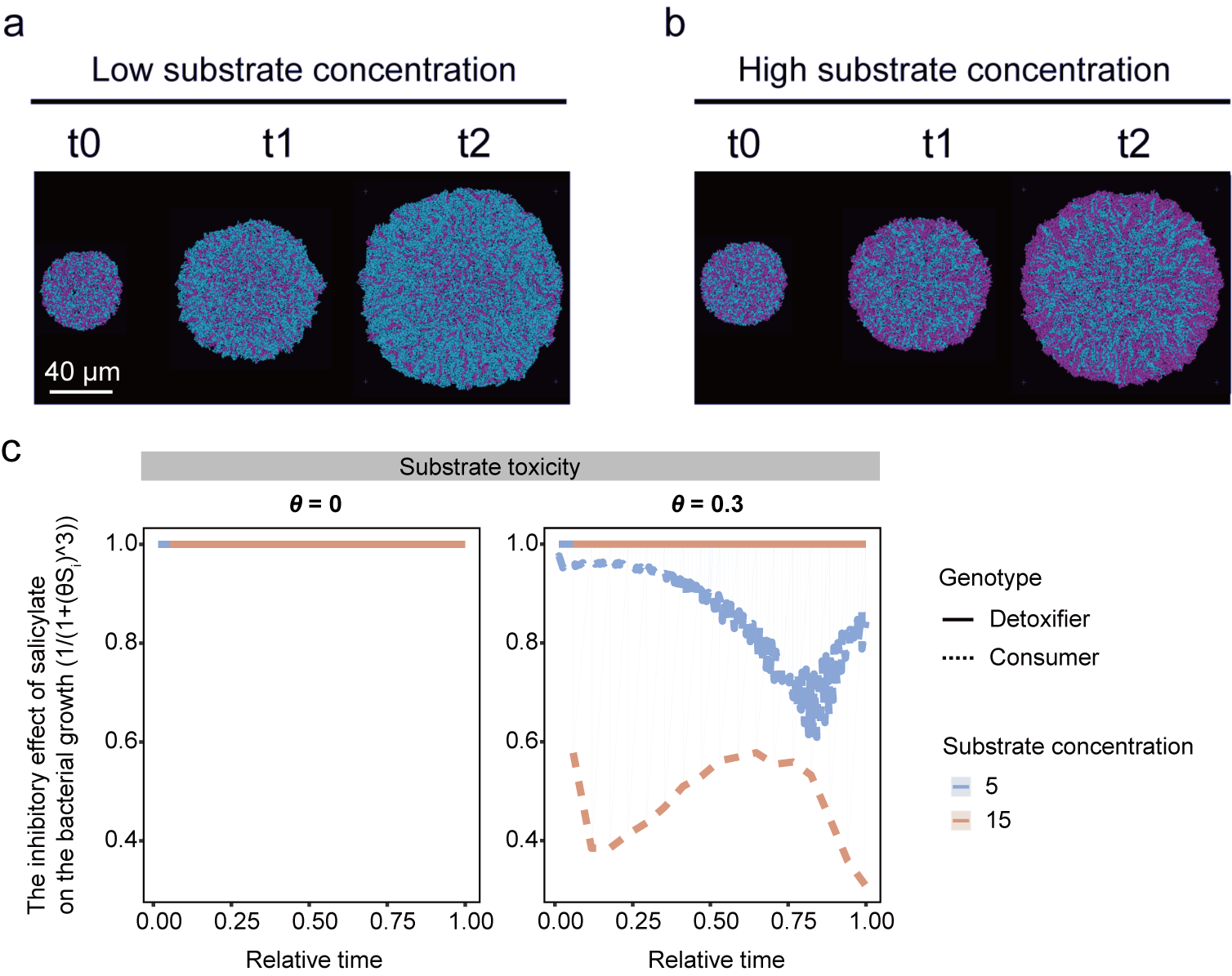
**

**Supplementary Figure 7** Spatial distribution of the two-strain cross-feeding community during colony expansion by computational modeling. In the colony images, detoxifier cells are shown in magenta, while consumer cells are shown in cyan. (a) Substrate does not have toxicity on bacteria. (b) The substrate has toxicity to bacteria. The detoxifier cells were pushed ahead by the proliferating consumer cells and dominated the colony periphery, which resulted in successive range expansion. (c) The inhibitory effect of salicylate on the growth of both the detoxifier and the consumer. Hill equation is used to model the effect of salicylate on bacterial growth rates ($\frac{\text{1}}{{\text{1+(θ}\text{s}_{i})}^{3}}$). In the absence of substrate toxicity ($\text{θ }\text{= 0}$), the value of Hill equation in both the detoxifier and the consumer is 1, which demonstrates that the salicylate has no effect on the growth of both the detoxifier and the consumer. In contrast, at a high substrate concentration of 15 C-mM, the value of Hill equation of the detoxifier is much higher than that of the consumer in the presence of substrate toxicity ($\text{θ}\text{ }\text{= 0.3}$), which demonstrates that salicylate has larger inhibitory effect on the growth of the consumer than the detoxifier.

**
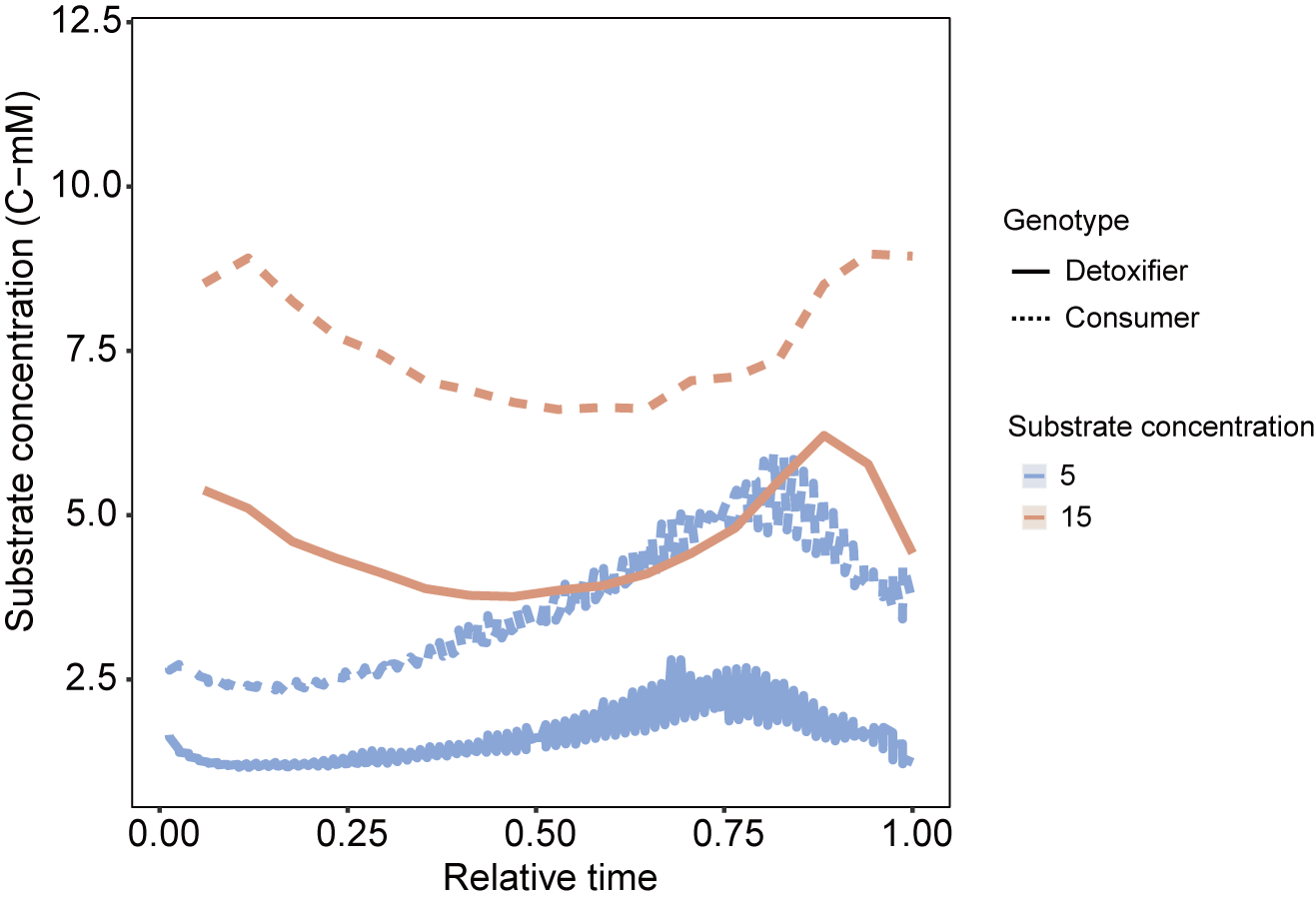
**

**Supplementary Figure 8** Dynamics of substrate distribution around the detoxifier and the consumer at the pattern edge during radial expansion in the presence of substrate toxicity ($\text{θ}\text{ }\text{= 0.3}$).

**
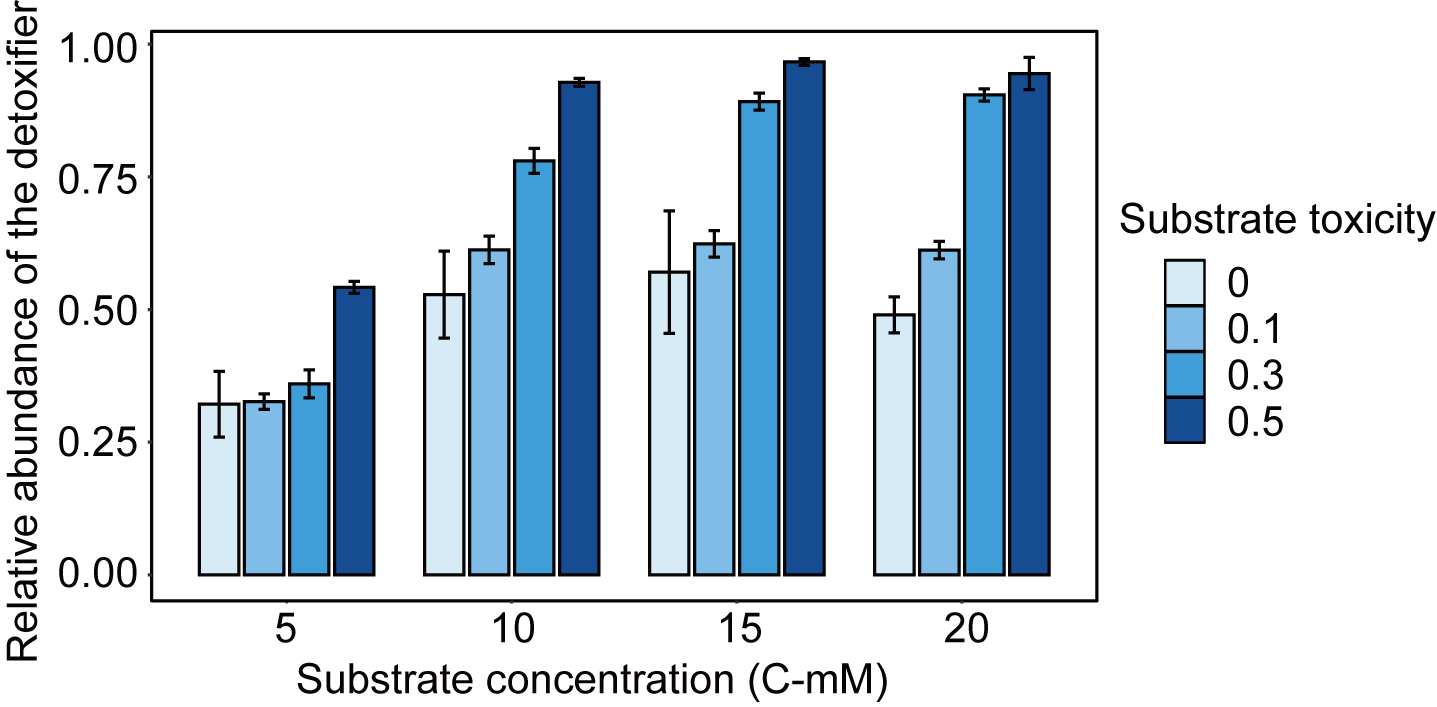
**

**Supplementary Figure 9** Effects of substrate toxicity and substrate concentration on detoxifier dominance at the expansion frontier in simulations. Increasing substrate toxicity improved the relative abundance of the detoxifier at the expansion frontier. At sufficiently high toxicity ($\theta$ = 0.5), the detoxifier dominated the expansion frontier even at relatively low substrate concentrations ($S$ = 10 C-mM), whereas higher substrate concentrations are required to achieve similar dominance at lower toxicity.

**
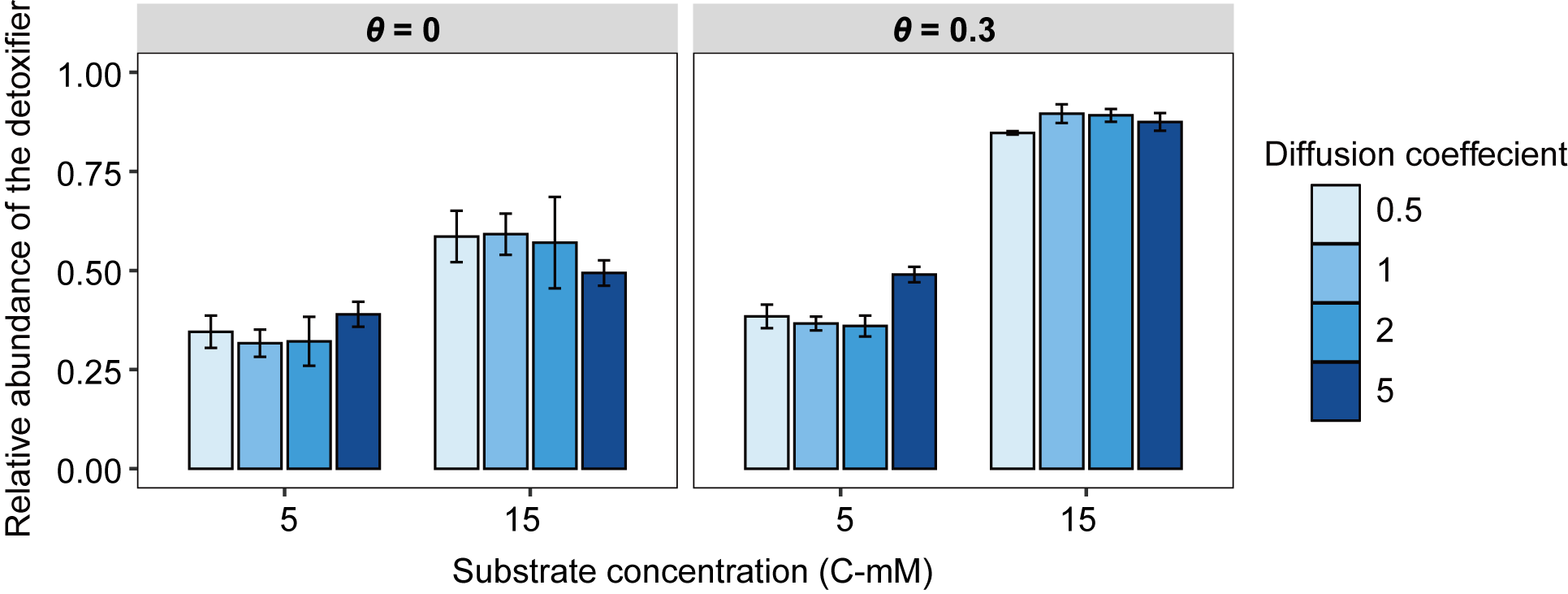
**

**Supplementary Figure 10** Effects of mass diffusion on relative abundance of the detoxifier at the expansion frontier in simulations. Results are shown for simulations conducted across a range of mass diffusion coefficients ($D =$ 0.5, 1, 2, and 5), both in the absence of substrate toxicity ($\theta$ = 0) and in the presence of substrate toxicity ($\theta$ = 0.3). In the absence of substrate toxicity, the relative abundance of the detoxifier at the expansion frontier remained low across all diffusion coefficients and substrate concentrations. When substrate toxicity was present, the detoxifier dominated the expansion frontier at 15 C-mM substrate across all diffusion coefficients.

**
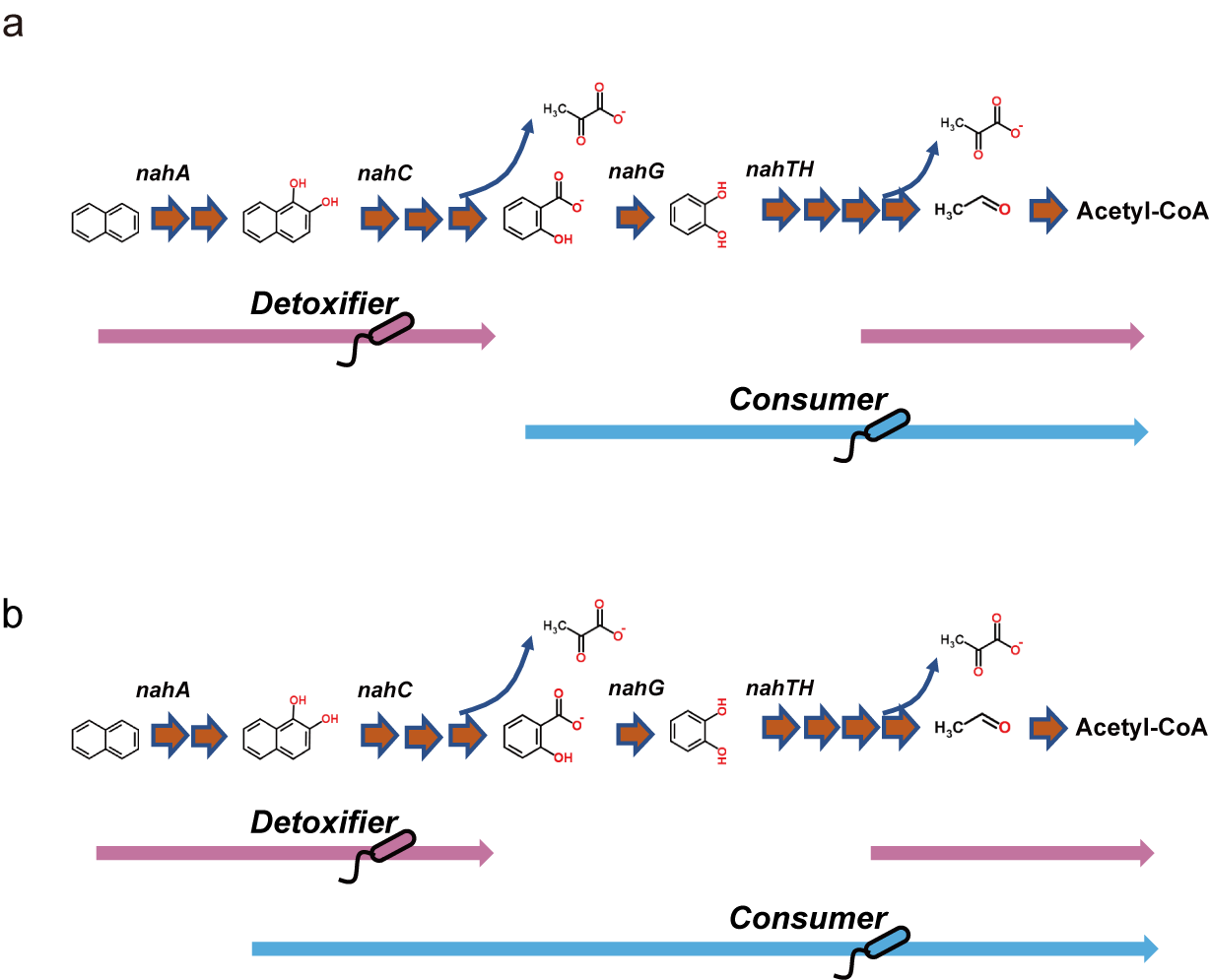
**

**Supplementary Figure 11** Synthetic microbial communities engaged in cross-feeding were used for naphthalene degradation in this study. The experimental system consists of isogenic mutants from *Pseudomonas stutzeri* AN10. Colored arrows (i.e., magenta and cyan) indicate the metabolic processes performed by each strain. (a) In the naphthalene degradation biosystem, the detoxifier contains two loss-of-function deletions in the *nahG* and *nahTH* and can catalyze naphthalene but not salicylate. The consumer contains two loss-of-function deletions in the *nahA* and *nahC* and can catalyze salicylate but not naphthalene. (b) In the naphthalene degradation biosystem, the detoxifier contains two loss-of-function deletions in the *nahG* and *nahTH* and can catalyze naphthalene but not salicylate. The consumer contains a single loss-of-function deletion in the *nahA* gene and can catalyze salicylate and 1,2-dihydroxynaphthalene but not naphthalene. In both (a) and (b), the detoxifier and the consumer display cooperation when grown together with an exogenous supply of naphthalene as the growth-limiting substrate. Definitions: nahA: naphthalene dioxygenase reductase; nahC: 1,2-dihydroxynaphthalene dioxygenase; nahG: salicylate hydroxylase; nahTH: catechol 2,3-dioxygenase.

**
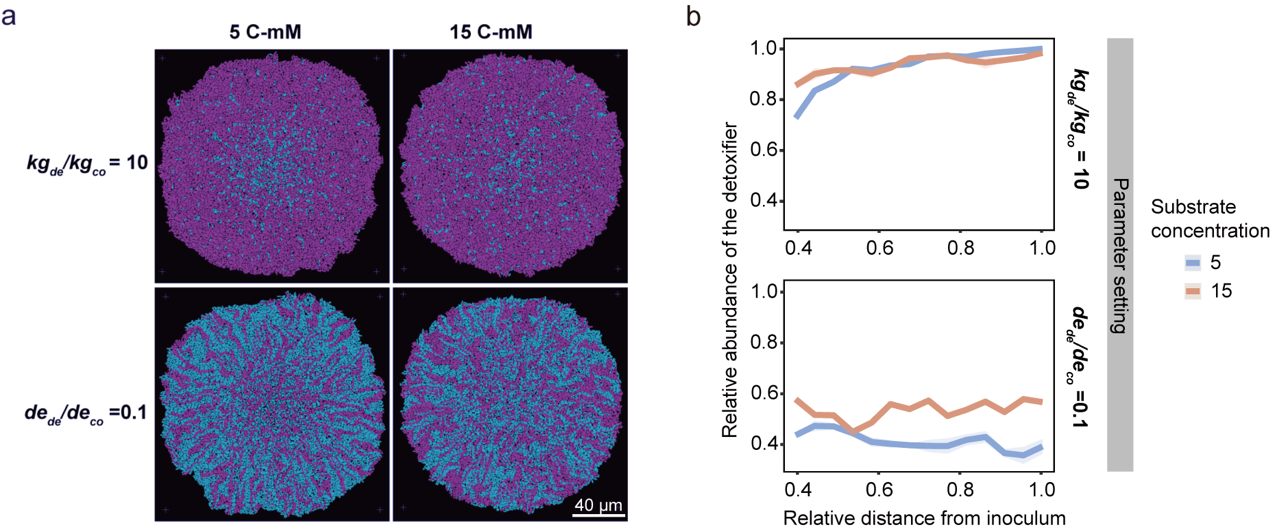
**

**Supplementary Figure 12** Computational modeling showed that setting growth rate differences between the two strains by altering ${kg}_{i}$ or $d_{i}$ could not result in pattern diversification in the absence of substrate toxicity. (a) Simulated images with a 10-fold difference in ${kg}_{i}$ was introduced between two strains (the detoxifier higher than the consumer, top row) or $d_{i}$ of the consumer was 10-fold higher than that of the detoxifier (bottom row) at the end of the simulation time. The final population size of the simulated colonies was approximately 8100 cells. Detoxifier cells are shown in magenta, while consumer cells are shown in cyan. (b) Measurement of relative abundance of the detoxifier from the edge of the inoculum towards the edge of expansion. Lines and shaded areas correspond to the mean of data points (n = 3) and 95% confidence interval of the mean, respectively.

**References**

1. Li H, Opgenorth PH, Wernick DG, Rogers S, Wu TY, Higashide W *et al.* Integrated electromicrobial conversion of co_2_ to higher alcohols. *Science*. 2012;**335**:1596 <https://doi.org/10.1126/science.1217643>

2. Liang J-L, Nie Y, Wang M, Xiong G, Wang Y-P, Maser E *et al.* Regulation of alkane degradation pathway by a tetr family repressor via an autoregulation positive feedback mechanism in a gram-positivedietziabacterium. *Mol Microbiol*. 2016;**99**:338-59 <https://doi.org/10.1111/mmi.13232>

3. Wang M, Chen X, Ma Y, Tang YQ, Johnson DR, Nie Y *et al.* Type iv pilus shapes a ‘bubble-burst' pattern opposing spatial intermixing of two interacting bacterial populations. *Microbiology Spectrum*. 2022;**10**:e0194421 <https://doi.org/10.1128/spectrum.01944-21>

4. Ozgen VC, Kong W, Blanchard AE, Liu F, Lu T. Spatial interference scale as a determinant of microbial range expansion. *Science Advances*. 2018;**4**:eaau0695 <https://doi.org/10.1126/sciadv.aau0695>

5. Ruan C, Ramoneda J, Gogia G, Wang G, Johnson DR. Fungal hyphae regulate bacterial diversity and plasmid-mediated functional novelty during range expansion. *Current Biology*. 2022;**32**:5285-94.e4 <https://doi.org/10.1016/j.cub.2022.11.009>

6. Goldschmidt F, Regoes RR, Johnson DR. Successive range expansion promotes diversity and accelerates evolution in spatially structured microbial populations. *ISME J*. 2017;**11**:2112-23 <https://doi.org/10.1038/ismej.2017.76>

7. Wang M, Chen X, Tang YQ, Nie Y, Wu XL. Substrate availability and toxicity shape the structure of microbial communities engaged in metabolic division of labor. *mLife*. 2022;**1**:131-45 <https://doi.org/10.1002/mlf2.12025>

8. Wang M, Chen X, Liu X, Fang Y, Zheng X, Huang T *et al.* Even allocation of benefits stabilizes microbial community engaged in metabolic division of labor. *Cell Rep*. 2022;**40**:111410 <https://doi.org/10.1016/j.celrep.2022.111410>

9. Camara B, Bielecki P, Kaminski F, dos Santos VM, Plumeier I, Nikodem P *et al.* A gene cluster involved in degradation of substituted salicylates via ortho cleavage in pseudomonas sp strain mt1 encodes enzymes specifically adapted for transformation of 4-methylcatechol and 3-methylmuconate. *J Bacteriol*. 2007;**189**:1664-74 <https://doi.org/10.1128/Jb.01192-06>

10. Zimmerman SB, Trach SO. Estimation of macromolecule concentrations and excluded volume effects for the cytoplasm of escherichia coli. *J Mol Biol*. 1991;**222**:599-620 <https://doi.org/10.1016/0022-2836(91)90499-v>

11. Viggiani A, Siani L, Notomista E, Birolo L, Pucci P, Di Donato A. The role of the conserved residues his-246, his-199, and tyr-255 in the catalysis of catechol 2,3-dioxygenase from pseudomonas stutzeri ox1. *Journal of Biological Chemistry*. 2004;**279**:48630-39 <https://doi.org/10.1074/jbc.M406243200>
